# Solenodon genome reveals convergent evolution of venom in eulipotyphlan mammals

**DOI:** 10.1073/pnas.1906117116

**Published:** 2019-11-26

**Authors:** Nicholas R. Casewell, Daniel Petras, Daren C. Card, Vivek Suranse, Alexis M. Mychajliw, David Richards, Ivan Koludarov, Laura-Oana Albulescu, Julien Slagboom, Benjamin-Florian Hempel, Neville M. Ngum, Rosalind J. Kennerley, Jorge L. Brocca, Gareth Whiteley, Robert A. Harrison, Fiona M. S. Bolton, Jordan Debono, Freek J. Vonk, Jessica Alföldi, Jeremy Johnson, Elinor K. Karlsson, Kerstin Lindblad-Toh, Ian R. Mellor, Roderich D. Süssmuth, Bryan G. Fry, Sanjaya Kuruppu, Wayne C. Hodgson, Jeroen Kool, Todd A. Castoe, Ian Barnes, Kartik Sunagar, Eivind A. B. Undheim, Samuel T. Turvey

**Affiliations:** ^a^Centre for Snakebite Research & Interventions, Liverpool School of Tropical Medicine, Pembroke Place, L3 5QA Liverpool, United Kingdom;; ^b^Institut für Chemie, Technische Universität Berlin, 10623 Berlin, Germany;; ^c^Collaborative Mass Spectrometry Innovation Center, University of California, San Diego, La Jolla, CA 92093;; ^d^Department of Biology, University of Texas at Arlington, Arlington, TX 76010;; ^e^Department of Organismic and Evolutionary Biology, Harvard University, Cambridge, MA 02138;; ^f^Museum of Comparative Zoology, Harvard University, Cambridge, MA 02138;; ^g^Evolutionary Venomics Lab, Centre for Ecological Sciences, Indian Institute of Science, 560012 Bangalore, India;; ^h^Department of Biology, Stanford University, Stanford, CA 94305;; ^i^Department of Rancho La Brea, Natural History Museum of Los Angeles County, Los Angeles, CA 90036;; ^j^Institute of Low Temperature Science, Hokkaido University, 060-0819 Sapporo, Japan;; ^k^School of Life Sciences, University of Nottingham, University Park, NG7 2RD Nottingham, United Kingdom;; ^l^Biomedical Research Centre, University of East Anglia, Norwich Research Park, NR4 7TJ Norwich, United Kingdom;; ^m^Ecology and Evolution Unit, Okinawa Institute of Science and Technology, Onna, Kunigami-gun, Okinawa, 904-0495, Japan;; ^n^Division of BioAnalytical Chemistry, Amsterdam Institute of Molecules, Medicines and Systems, Vrije Universiteit Amsterdam, 1081 LA Amsterdam, The Netherlands;; ^o^Durrell Wildlife Conservation Trust, Les Augrès Manor, Trinity, Jersey JE3 5BP, British Channel Islands, United Kingdom;; ^p^SOH Conservación, Apto. 401 Residencial Las Galerías, Santo Domingo, 10130, Dominican Republic;; ^q^Venom Evolution Lab, School of Biological Sciences, University of Queensland, St. Lucia, QLD 4067, Australia;; ^r^Naturalis Biodiversity Center, 2333 CR Leiden, The Netherlands;; ^s^Vertebrate Genomics, Broad Institute of MIT and Harvard, Cambridge, MA 02142;; ^t^Program in Bioinformatics and Integrative Biology, University of Massachusetts Medical School, Worcester, MA 01655;; ^u^Science for Life Laboratory, Department of Medical Biochemistry and Microbiology, Uppsala University, 751 23 Uppsala, Sweden;; ^v^Monash Venom Group, Department of Pharmacology, Biomedicine Discovery Institute, Monash University, VIC 3800, Australia;; ^w^Department of Biochemistry & Molecular Biology, Biomedicine Discovery Institute, Monash University, VIC 3800, Australia;; ^x^Department of Earth Sciences, Natural History Museum, SW7 5BD London, United Kingdom;; ^y^Centre for Advanced Imaging, The University of Queensland, Brisbane QLD 4072, Australia;; ^z^Institute for Molecular Bioscience, The University of Queensland, Brisbane QLD 4072, Australia;; ^aa^Centre for Ecological and Evolutionary Synthesis, Department of Biosciences, University of Oslo, Oslo 0316, Norway;; ^bb^Institute of Zoology, Zoological Society of London, Regent’s Park, NW1 4RY London, United Kingdom

**Keywords:** convergent molecular evolution, genotype phenotype, gene duplication, venom systems, kallikrein toxin

## Abstract

Venom systems are key adaptations that have evolved throughout the tree of life and typically facilitate predation or defense. Despite venoms being model systems for studying a variety of evolutionary and physiological processes, many taxonomic groups remain understudied, including venomous mammals. Within the order Eulipotyphla, multiple shrew species and solenodons have oral venom systems. Despite morphological variation of their delivery systems, it remains unclear whether venom represents the ancestral state in this group or is the result of multiple independent origins. We investigated the origin and evolution of venom in eulipotyphlans by characterizing the venom system of the endangered Hispaniolan solenodon (*Solenodon paradoxus*). We constructed a genome to underpin proteomic identifications of solenodon venom toxins, before undertaking evolutionary analyses of those constituents, and functional assessments of the secreted venom. Our findings show that solenodon venom consists of multiple paralogous kallikrein 1 (*KLK1*) serine proteases, which cause hypotensive effects in vivo, and seem likely to have evolved to facilitate vertebrate prey capture. Comparative analyses provide convincing evidence that the oral venom systems of solenodons and shrews have evolved convergently, with the 4 independent origins of venom in eulipotyphlans outnumbering all other venom origins in mammals. We find that *KLK1*s have been independently coopted into the venom of shrews and solenodons following their divergence during the late Cretaceous, suggesting that evolutionary constraints may be acting on these genes. Consequently, our findings represent a striking example of convergent molecular evolution and demonstrate that distinct structural backgrounds can yield equivalent functions.

## Significance

Multiple representatives of eulipotyphlan mammals (shrews, hedgehogs, moles, and solenodons) are venomous, but little is known about the evolutionary history and composition of their oral venom systems. Herein we characterized venom from the endangered Hispaniolan solenodon (*Solenodon paradoxus*) and find that it consists of hypotensive proteins likely used to facilitate vertebrate prey capture. We demonstrate that venom has evolved independently on at least 4 occasions in eulipotyphlans, and that molecular components of these venoms have also evolved convergently, with kallikrein-1 proteins coopted as toxins in both solenodons and shrews following their divergence over 70 million years ago. Our findings present an elegant example of convergent molecular evolution and highlight that mammalian venom systems may be subjected to evolutionary constraints.

Venom systems are key ecological innovations that have evolved independently on numerous occasions across the tree of life (1). They consist of mixtures of proteinaceous components (commonly referred to as toxins) and can be defined as secretions produced in specialized tissues that cause physiological perturbations when delivered into other animals through a wound caused by a venom delivery apparatus (2). Venoms have proven to be valuable systems for understanding a variety of different evolutionary processes, including those relating to convergence (1, 2), accelerated molecular evolution (3), gene duplication (4), and protein neofunctionalization (5). Venoms are also of great medical importance, both due to the harm they can cause to people (e.g., >100,000 people die annually as a result of snake envenoming) (6) and for the value of their highly selective toxins for understanding physiological processes and the development of new pharmaceuticals (7).

Ecologically, venoms are primarily used for prey capture and/or to defend the producing animal from aggressors or predators, though in some instances venom is utilized for intraspecific competition or to facilitate offspring survival (1). Despite extensive research focus on a number of venomous lineages, many remain almost completely unstudied, including venomous mammals. Mammalian venom systems are rare and, based on the definition above, are restricted to members of 4 extant orders: the monotremes, chiropterans, primates, and eulipotyphlans (8). Their venoms are utilized for distinct ecological purposes, such as male–male combat to facilitate breeding (platypus; *Ornithorhynchus anatinus*), aiding hematophagy (vampire bats; *Desmodus rotundus*, *Diaemus youngi*, and *Diphylla ecaudata*), predation (shrews; *Blarina brevicauda*, *Crocidura canariensis*, *Neomys fodiens*, and *Neomys anomalus*), and potentially defensive or antagonistic purposes (slow lorises; *Nycticebus* spp.) (8).

**Fig. 1.**
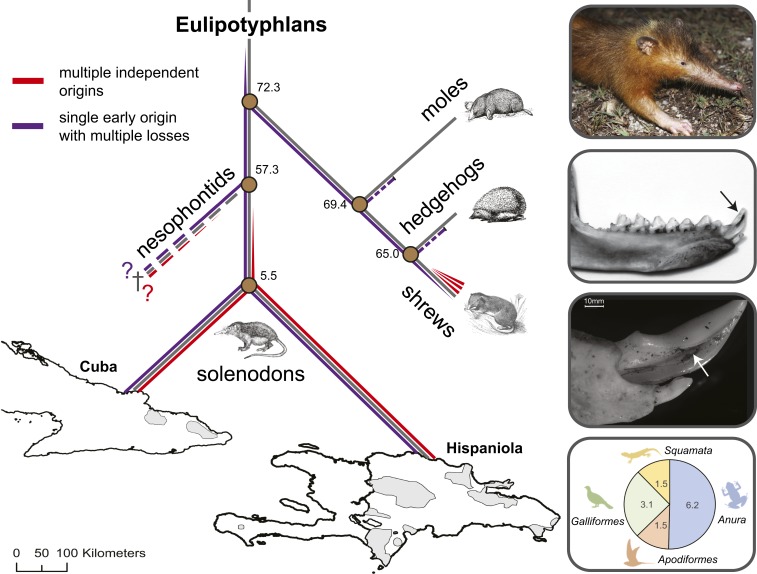
The 2 competing hypotheses relating to the origin of venom in eulipotyphlans and key characteristics of the Hispaniolan solenodon (*S. paradoxus*). The schematic phylogeny (gray lines) highlights the estimated divergence times of eulipotyphlan families and solenodon species and the 2 competing hypotheses relating to the origin of venom in this group. Purple lines indicate the early origin of venom hypothesis followed by losses in moles, hedgehogs, and some shrews (narrowing line), whereas red lines indicate the alternative hypothesis of multiple independent origins of venom in shrews (3 times), solenodons, and possibly nesophontids. Shaded areas of the map indicate the modern distribution of the 2 solenodon species (*A. cubana* and *S. paradoxus*) on the islands of Cuba and Hispaniola, respectively. Boxes on the *Right* show (from *Top* to *Bottom*): a wild specimen of *S. paradoxus*, its lower jaw morphology, its enlarged tubular lower second incisor used for venom delivery visualized via stereo microscopy, and the composition and frequency of occurrence (in percentages) of vertebrates detected in their diet determined by DNA barcoding analyses of fecal samples. Divergence times displayed on the phylogeny are from refs. 15 and 19. The photograph of the wild solenodon is courtesy of Rocio Pozo.

The Eulipotyphla, a group historically referred to as the insectivores, consists of the hedgehogs, moles, shrews, and solenodons. Within this group, species from 3 separate genera of shrews (*Blarina*, *Neomys*, and *Crocidura*) and the solenodons (*Solenodon paradoxus* and *Atopogale cubana*) exhibit convincing evidence of an oral venom system (9) (Fig. 1). Shrews utilize their venom for overpowering vertebrate prey much larger than they would otherwise be able to feed upon (e.g., similar mass to themselves) and for paralyzing invertebrate prey for long-term storage purposes (“prey caching”), presumably to provide a continual resource to help offset the extreme metabolic demands of these small animals (10–13). While convincing evidence of a venom system is lacking for hedgehogs (14) and moles, it has been proposed that the nesophontids (*Nesophontes* spp.), a recently extinct family of eulipotyphlans that were the sister group to the solenodons (15), may also have been venomous based on morphological evidence (16). All of the extant venomous eulipotyphlans produce venom in submaxillary glands, but their venom delivery apparatus varies, with solenodons using elaborate tubular lower incisors (Fig. 1) and shrews having little-modified but pointed lower incisors and canines (9, 17). While this morphological variation might point toward independent origins of these venom systems, with the more extensive morphological adaptation in solenodons potentially indicating a longer evolutionary history or tighter ecological integration of venom use, it is worth noting that the venom-delivering dentition of snakes also varies extensively, despite the common origin of their venom secretions and toxins (18). Thus, it remains unclear whether eulipotyphlan venom systems share a single early evolutionary origin, or whether multiple groups of shrews, the solenodons, and possibly other eulipotyphlans have each evolved venom independently following their divergence during the Late Cretaceous Period.

To address this fundamental question, we characterized the venom system of the Hispaniolan solenodon (*S. paradoxus*). Solenodons are relatively large (∼1 kg) nocturnal eulipotyphlans with diagnostic grooved caniniform second lower incisors. They are found on the Caribbean islands of Hispaniola (*S. paradoxus*) and Cuba (*A. cubana*), and molecular and fossil evidence suggests that they diverged from all other mammals over 70 million years ago (MYA) (19) (Fig. 1). Both species have long been considered rare and threatened and have experienced range declines associated with habitat loss and predation by invasive dogs and cats (20). Despite these enigmatic animals likely being the largest extant venomous terrestrial mammals, little is known about the composition, function, and ecological role of their venom, other than its relatively weak toxicity to mice (17). Consequently, we sequenced the genome of the Hispaniolan solenodon and used this information to underpin identifications of the proteins present in its venom. We then characterized the function of solenodon venom via a range of in vitro and in vivo assays to determine the likely role of this adaptation. Our findings reveal that eulipotyphlan venom systems and their constitutive toxins have evolved on multiple independent occasions via the process of convergent evolution.

## Results and Discussion

We constructed a genome for *S. paradoxus* from DNA isolated from blood collected from an adult male Hispaniolan solenodon from the northern Dominican Republic (*S. p. paradoxus*), housed in captivity in the Dominican Republic National Zoo (ZOODOM). DNA was sequenced using Illumina paired-end short-read technology, and the genome was assembled using DISCOVAR de novo. The resulting assembly (21) had a scaffold N50 of 407.7 kb and performed well on benchmarking universal single-copy orthologs (BUSCO) (22), with 92.9% complete and 4.7% partial BUSCOs recovered. The assembly is thus relatively higher quality than a recently published “consensus” genome for *Solenodon p. woodi* constructed using DNA from multiple individuals (23) (*SI Appendix*, Table S1). Next, we annotated the repetitive and protein-coding portions of the genome using MAKER (24). Because RNA-sequencing (RNA-seq) data were not available for this endangered species, our annotations were based on homology searches alone, which may be less effective for identifying highly divergent genes. Nonetheless, homology searches with existing protein databases and genome and RNA-seq data from related eulipotyphlan species (hedgehog, mole, shrew) identified a comparably high number of protein-encoding genes (18,112 vs. 19,372 to 20,798), of which the vast majority exhibited orthology with those previously detected from other eulipotyphlans (97.4 to 98.0%), indicating that our approach was broadly effective.

**Fig. 2.**
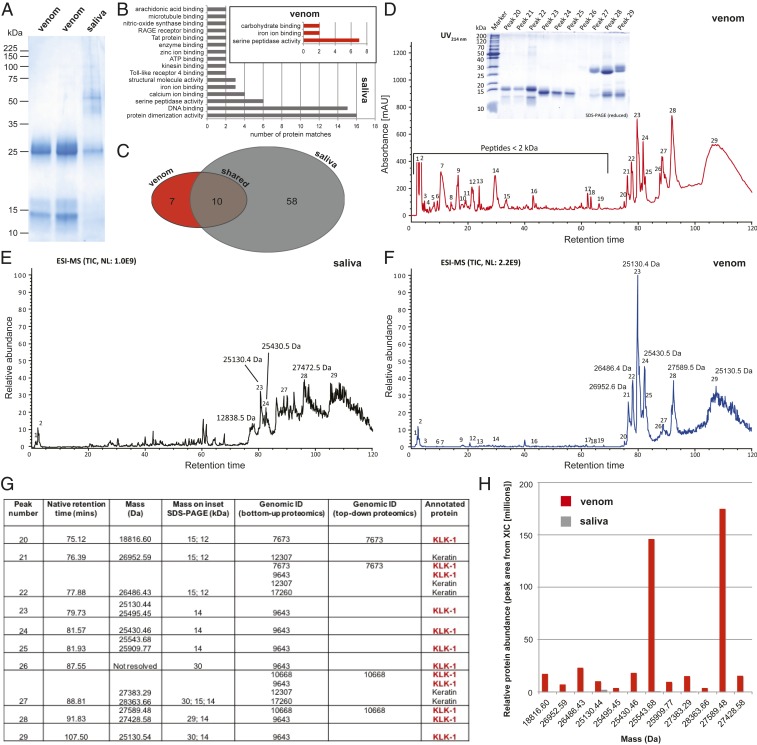
Proteomic analyses of Hispaniolan solenodon (*S. paradoxus*) venom and saliva reveal *KLK1* proteins as major venom components. (*A*) Reduced SDS-PAGE gel electrophoretic profiles of venom and saliva samples. (*B*) Gene ontology (GO) term analysis of proteins identified via shotgun proteomic-based annotation to the genome. GO term categories are only displayed for those with at least 2 matches. (*C*) Venn diagram displaying the number of proteins in the venom and saliva, and those identified in both samples via shotgun proteomic-based annotation to the genome. (*D*) Reverse-phase chromatographic separation of venom. Venom was separated by semipreparative reversed-phase HPLC (UV_214nm_) and manually collected. Peptides were directly submitted to LC-MS/MS, whereas protein fractions were analyzed by SDS-PAGE (*Inset*) under reducing conditions. Afterward, protein bands were subjected to in-gel trypsin digestion and identified by spectrum peptide matching against the translated *S. paradoxus* genome database. (*E* and *F*) LC-top-down MS analysis of saliva (*E*) and venom (*F*). The peak nomenclature is based on the chromatogram fractions, shown in *D*. (*E*) Total ion current (TIC) profile of native saliva separated by HPLC. (*F*) TIC profile of native venom separated by HPLC. (*G*) Summary table of the proteins identified via top-down and bottom-up proteomic analyses of solenodon venom, including their mass, corresponding identification in the genome (genome ID), and protein annotation. All identified proteins are annotated as *KLK1*, with the exception of keratins, which are human contaminants. (*H*) Comparison of the relative abundance of main proteins present in chromatographic fractions of venom and saliva from top-down MS experiments. *SI Appendix*, Table S2 presents a summary of the protein matches identified in solenodon venom and saliva via the various proteomic approaches.

Venom was collected from 2 wild male adult Hispaniolan solenodons (*S. p. woodi*) that were caught near Pedernales, southwestern Dominican Republic, and we also collected saliva from 1 of these individuals. Saliva was collected via direct pipetting from the back of the mouth prior to venom stimulation, while venom was collected by encouraging solenodons to chew onto soft plastic tubing and collecting the resulting secretions. Thus, saliva is unlikely to contain venom proteins, but venom may, perhaps, contain small amounts of salivary proteins. However, initial 1D SDS-PAGE gel electrophoretic analysis of these samples validated the collection approach, as distinct protein profiles were observed between the collected venom and saliva (Fig. 2*A*). We also observed highly similar venom compositions between the 2 sampled individuals (Fig. 2*A*), suggesting venom conservation. However, the small sample size and possibility of high genetic relatedness of these individuals means that future work is required to robustly explore venom variation in solenodons. For in-depth comparisons between venom and saliva, we applied 3 different mass spectrometry-based proteomics workflows: shotgun analyses of digested crude samples, bottom-up proteomic analyses of prefractionated (decomplexed) samples, and top-down proteomic analyses of reduced and nonreduced samples. In addition to orthogonal confirmation of the main venom components, the application of these 3 different approaches offers complementary merits such as higher sensitivity, optimal quantitative estimation of toxin abundance, and proteoform-resolved compositional information, respectively. For all approaches, venom proteins were identified by peptide/protein spectrum matching against the protein database derived from the assembled *S. paradoxus* genome.

Initial analysis via shotgun experiments revealed solenodon venom is primarily composed of proteins that exhibit high-scoring annotations to kallikrein-1-like serine proteases (*KLK1*-like; 7 of 17 total venom proteins identified), although various other protein types were also detected (Fig. 2 *B* and *C* and *SI Appendix*, Table S2). None of the venom proteins directly identified here show similarity to those recently predicted by other researchers, who used genomic data alone to predict venom toxin identity based on sequence similarity to previously described, yet distinct, animal venom toxins (23). These findings highlight the importance of direct sampling (e.g., gene expression or protein) to robustly characterize proteins associated with venom secretions (25). The majority (10 proteins) of the solenodon venom proteins detected were also identified in saliva, although solenodon saliva contained an additional 48 proteins with diverse functional annotations (Fig. 2 *B* and *C* and *SI Appendix*, Table S2). Next, we applied a validated venom decomplexation strategy that utilized high-performance liquid chromatography (HPLC) fractionation followed by SDS-PAGE, in-gel trypsin digestion, and liquid chromatography-tandem mass spectrometry (LC-MS/MS) analysis (26, 27). This approach yielded 29 venom peaks (Fig. 2*D*), with peaks 1 through 19 containing molecules with masses below 3 kDa, peaks 20 through 25 showing masses of 10 to 15 kDa according to reductive SDS-PAGE, and peaks 27 through 29 showing 2 masses around 14 and 28 kDa. From these excised bands (14 and 28 kDa), we identified 3 distinct *KLK1*-like proteins (Fig. 2*G* and *SI Appendix*, Table S2), and no other proteins, with the exception of keratin contaminants. We complemented these data with top-down analyses of crude reduced and nonreduced venom and saliva. In this experiment, the venom and saliva were not digested and were instead directly analyzed by LC-MS/MS, which allows better comparison of homologous proteins and proteoforms that would otherwise be indistinguishable after trypsin digestion (28). According to the UV peak area, the main protein observed in the native (nonreduced) venom was found in peak 28 with the monoisotopic mass 27589.64 Da and a retention time (RT) of 92.3 min, and which again corresponded to *KLK1* (Fig. 2*F*). This protein was not detected in the saliva from the in-gel digest (Fig. 2 *E* and *H*). Several proteins in the same mass range (26952.59 Da, 76.4 min; 26486.43 Da, 77.9 min; 25430.46 Da, 81.6 min; 27589.48 Da, 91.83 min; and 25130.54 Da, 107.5 min) were also mainly detected in the venom. Another high abundance *KLK1*-like isoform with a mass of 25130.40 Da and RT of 80.5 min was detected in both saliva and venom, although its relative abundance (normalized peak area) was around 5-fold higher in the venom (Fig. 2 *E*–*H*).

**Fig. 3.**
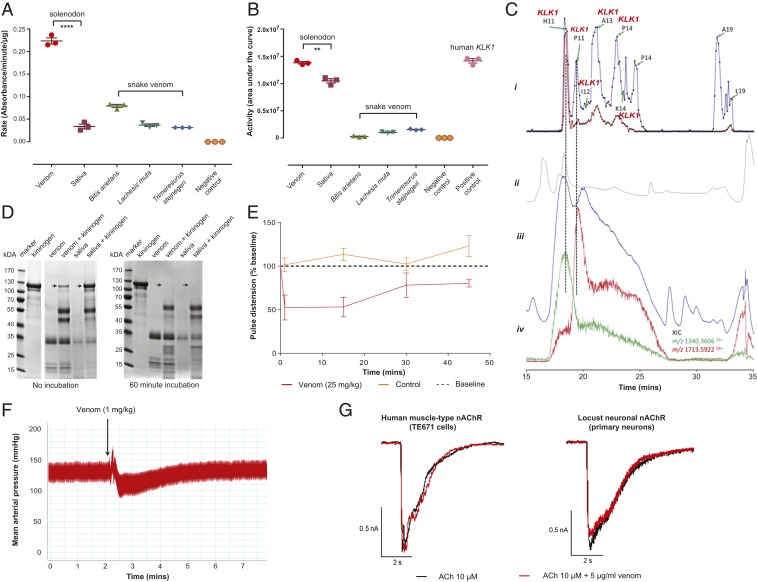
Functional assessments of Hispaniolan solenodon (*S. paradoxus*) venom reveals kallikrein serine protease activity and hypotensive effects. Solenodon venom has extensive (*A*) serine protease activity, as measured by chromogenic enzyme assay, and (*B*) plasminogen-activating activity, as measured by fluorescent enzyme assay. The data displayed are the mean rate of substrate consumption (*A*) or area under the kinetic curve (*B*) for mean measurements (±SEM) taken from 3 independent experiments; ****P < 0.0001; **P < 0.01; unpaired 2-tailed *t* tests. (*C*) Nanofractionation bioassaying reveals that *KLK1* proteins are responsible for plasminogen-activating activity. (*i*) Bioactivity chromatogram at 5 mg/mL (blue line) and 1 mg/mL (red line) venom show the activity of each fraction, where positive peaks represent bioactive compounds. Bioactive wells selected for tryptic digestion are indicated by green arrows and well numbers, and those identified by mass spectrometry as *KLK1*s are labeled in red. (*ii*) UV trace at 254 nm collected during the LC-MS run with a UV-visible spectroscopy detector. (*iii*) TIC shown by the LC-MS chromatogram. (*iv*) Extracted ion currents (XICs) of the *m/z* values from the LC-MS data corresponding to the bioactives detected in the plasminogen assay. (*D*) Solenodon venom degrades high molecular weight kininogen more potently than saliva without incubation. SDS-PAGE gel electrophoresis profiles demonstrate that both venom and saliva completely degrade kininogen (arrows) when preincubated for 60 min, but that venom also degrades kininogen in the absence of preincubation. (*E*) Solenodon venom causes substantial reductions in the pulse distension of envenomed mice (25 mg/kg; *n* = 3) when compared to baseline measurements and controls (saline; *n* = 3). The data displayed represent mean measurements, and the error bars represent SDs. (*F*) Solenodon venom causes a transient depressor effect on the mean arterial blood pressure of the anesthetized rat. The data displayed are a representative trace from 1 of 5 experimental animals that received 1 mg/kg venom (see also *SI Appendix*, Fig. S3). (*G*) Solenodon venom has no effect on nicotinic acetylcholine receptors. Representative whole-cell patch-clamp traces showing human muscle-type TE671 (*Left*) and locust neuron nAChR (*Right*) responses to 10 µM acetylcholine and the coapplication of acetylcholine with 5 µg/mL solenodon venom. V_H_ = −75 mV.

These proteomic data demonstrate that: 1) solenodon venom is relatively compositionally streamlined in comparison with saliva; 2) venom consists predominately of *KLK1*-like proteins; and 3) while some of these *KLK1*-like proteins are also found in solenodon saliva, they are of much higher abundance in venom. Kallikreins are members of the S1 group of serine proteases and likely originated in early tetrapods (29, 30). They are diverse in placental mammals, consisting of up to 15 paralogs, and they act by enzymatically cleaving peptide bonds (29, 30). Kallikreins can have diverse functions, including cleaving kininogens and plasminogen, resulting in the liberation of kinins and plasmin, respectively (30). Here we demonstrate that solenodon venom exhibits activities consistent with the presence of secretions rich in kallikreins. Using substrate-specific kinetic biochemical assays, we find that solenodon venom exhibits serine protease activity and potently activates plasminogen (Fig. 3 and *SI Appendix*, Fig. S1). In both cases, solenodon venom showed significant increases in activity when compared with solenodon saliva, and also when compared with snake venoms known to exert serine protease and plasminogen activating activities (Fig. 3 *A* and *B*) (31, 32). We demonstrated that multiple *KLK1*-like proteins are responsible for the activation of plasminogen observed with solenodon venom via the use of a nanofractionation approach consisting of LC-MS, undertaken in parallel with a specific bioassay (Fig. 3*C* and *SI Appendix*, Table S3). Both venom and saliva also demonstrated cleavage of high molecular weight kininogen (HMWK), with the venom being most potent, as it rapidly cleaved this substrate in the absence of preincubation, unlike saliva (Fig. 3*D*). While both venom and saliva were also found to cleave other substrates known to be targeted by serine proteases (e.g., fibrinogen) (*SI Appendix*, Fig. S1), their higher potency to HMWK is consistent with the identification of *KLK1* in these samples. In combination, these in vitro bioactivity studies reveal that solenodon venom exhibits functional specificities consistent with the identification of kallikrein serine proteases as the most numerous and abundant proteins found in the venom.

Physiologically, the cleavage of kininogens by kallikreins results in the liberation of the kinins bradykinin and kallidin, which in turn stimulate hypotensive responses in vertebrates, via the kinin–kallikrein system (30). To test whether solenodon venom causes hypotension in vivo, we i.v. administered a sublethal dose of venom in PBS to mice (25 mg/kg; *n* = 3) and compared their physiological responses with those of a control group receiving PBS only (*n* = 3). Using a MouseOx pulse-oximeter cuff, we periodically monitored the pulse rate, respiration rate, and percentage oxygen content of the envenomed and control animals but found no significant differences between the 2 groups (*SI Appendix*, Fig. S2). However, measures of pulse distension—defined as local blood flow at the sensor location—showed a substantial transient reduction in envenomed animals compared to controls (47.5% maximal decrease from baseline), with recovery toward baseline levels occurring 30 min after venom administration (Fig. 3*E*). These results suggest that solenodon venom exerts a hypotensive effect. To directly test this hypothesis, we assessed the bioactivity of solenodon venom in an in vivo cardiovascular assay. We found that solenodon venom (1 mg/kg; *n* = 5) caused a marked depressor effect on the mean arterial pressure of anesthetized rats, consisting of a transient depressor response and resulting in a maximal decrease of 22% (±6%) from baseline readings (Fig. 3*F* and *SI Appendix*, Fig. S3).

**Fig. 4.**
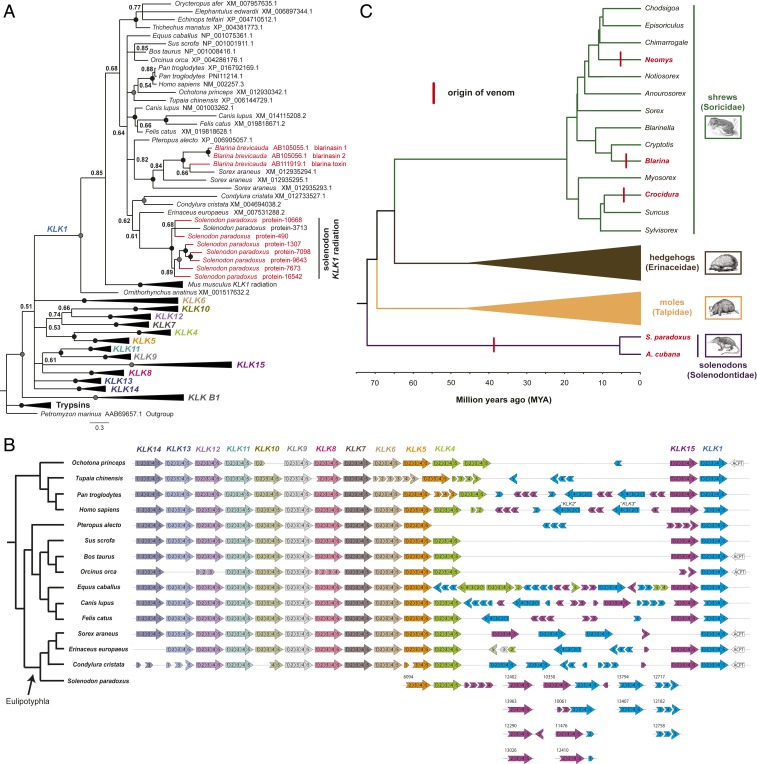
Molecular analyses reveal that eulipotyphlan venom systems and their toxin constituents have evolved independently by convergent evolution. (*A*) Molecular phylogeny of amino acid translations of tetrapod *KLK*s demonstrate that solenodon *KLK1* venom genes form a strongly supported monophyly and are polyphyletic to *Blarina* shrew venom genes. The phylogeny was derived by Bayesian inference analysis (*n* = 106; 2 × 10^8^ generations, 4 parallel runs with 6 simultaneous MCMC simulations). Genes encoding for proteins detected in solenodon venom (*SI Appendix*, Table S2) or *Blarina* venom (11, 33) are highlighted by red-colored branches and tip labels. Support values represent Bayesian posterior probabilities (BPP), where black circles represent BPP = 1.00 and gray circles BPP ≥ 0.95. See also *SI Appendix*, Fig. S4 for the nucleotide-derived phylogeny. (*B*) Analysis of the genomic organization of mammalian *KLK*s demonstrates that *KLK1*s are atypically numerous in the solenodon. Distinct patterns of *KLK1* orientation across eulipotyphlans suggest that venom genes have arisen independently in the solenodon, and evidence of multiple solenodon genome scaffolds containing *KLK1* and *KLK15* adjacent to one another suggests that these may form the basis of a duplication cassette. (*C*) Ancestral state reconstruction of the origin of venom in eulipotyphlans reveals that venom most likely evolved independently on 4 occasions (red vertical lines). Genera containing venomous species (or the species themselves) are highlighted by red tip labels. The computed ancestral traits for each node are depicted by pie charts, where the proportion of red color represents the posterior probability of the most recent common ancestor being venomous, and blue represents nonvenomous. In all cases, ancestral nodes support the nonvenomous character state with a posterior probability of 1.00, except for the *Suncus* and *Crocidura* node, where the support value was greater than 0.85. Divergence times are indicated by the scale, and these, along with the tree topology, are derived from prior studies (15, 19, 37). The specific timing of the origin of venom should not be inferred from the placement of the vertical red bars on the tree—these are placed arbitrarily at the midpoint of each relevant branch.

Our findings demonstrate that *KLK1*-like proteins are the major functional components of solenodon venom. S1 serine proteases are common constituents of animal venoms, with diverse venomous taxa such as snakes, lizards, cephalopods, and lepidopterans all utilizing representatives of this large multilocus gene family as toxins via the process of convergent evolution (2). Reconstructing the molecular evolutionary history of tetrapod kallikreins (*KLK1*–*KLK15*) (Fig. 4*A* and *SI Appendix*, Fig. S4) revealed that all of the annotated *KLK1*-like genes identified in the solenodon genome are indeed found nested within a strongly supported clade containing *KLK1*s from other mammals. Fascinatingly, this clade also includes proteins previously identified in the venom of the shrew *B. brevicauda* (blarina toxin and blarinasin 1 and 2) (11, 33) (Fig. 4*A*). However, the 7 *KLK1*s we identified in solenodon venom (*SI Appendix*, Table S2) formed a strongly supported monophyletic subcluster (Bayesian posterior probability: 1.00; bootstrap: 100), and included an additional solenodon *KLK1* isoform not identified by our proteomic analyses of venom (Fig. 4*A*). These findings strongly suggest that solenodon *KLK1* venom genes have arisen as the result of lineage-specific gene duplication events, rather than duplications occurring prior to the diversification of eulipotyphlans, thereby indicating independent venom-related diversifications in solenodons and shrews. To investigate this further, we performed sequence analyses of representative eulipotyphlan *KLK1*s. Prior work has suggested that a combination of multiple small insertions and alterations to the physicochemical patterns (hydropathicity and charge) of the 5 regulatory loops present in *KLK1*s are responsible for the increases in toxicity observed between blarina toxin and the blarinasins (34). Here, we find small insertions in the regulatory loops of solenodon venom *KLK1*s, although we find no consistent patterns of changes to the mean hydropathicity or charge of these regions when broadly comparing eulipotyphlan venom *KLK1*s with those identified from nonvenomous taxa (hydropathicity, *P* = 0.18; charge, *P* = 0.20) (*SI Appendix*, Fig. S5). However, comparisons of the locations of the regulatory loop insertions reveals a differential pattern between *Blarina* and *Solenodon*, with the former exhibiting insertions predominately in loops 1 and 2, and the latter in loop 3 (*SI Appendix*, Fig. S5), thereby confirming that these toxins have evolved independently for a role in venom.

Next, we employed site-, branch-, and branch-site-specific maximum likelihood and Bayesian models to assess the regime of natural selection influencing the evolution of the kallikrein gene family in tetrapods. Site-specific selection analyses (model 8, PAML [Phylogenetic analysis by maximum likelihood]) (35) revealed a significant influence of purifying selection on the evolution of all *KLK* paralogs. Each paralog was characterized by a very small omega (ω) value (mean *KLK* ω = 0.29), which represents the ratio of nonsynonymous-to-synonymous substitutions, with the exception of *KLK1* (ω = 0.55) (*SI Appendix*, Table S4 and Fig. S6). Our analyses identified 18 positively selected amino acid sites in *KLK1*, only 2 in *KLK10*, and none in any of the other kallikreins. These results suggest that while the majority of amino acid sites in *KLK1* remain extremely well conserved, a number have experienced positive selection for amino acid replacements. When overlaying these positively selected sites onto the *KLK1* sequence alignment, we find that 14 of these 18 sites are found within the 5 regulatory loops (*SI Appendix*, Fig. S5), which is consistent with the prior suggestion that modification of these regions may be important for venom toxin function (34). The findings of the site-specific selection analyses are further supported by Fast Unconstrained Bayesian AppRoximation (FUBAR) and mixed effects model evolution (MEME) analyses, which identified numerous *KLK1* sites evolving under the pervasive influence of purifying selection, but with only a small number evolving under pervasive or episodic positive selection (*SI Appendix*, Table S4). To identify whether positive selection has shaped the evolution of venom *KLK1*s detected in eulipotyphlans, we employed branch- and branch-site-specific maximum likelihood and Bayesian models. Together, these analyses revealed an increased influence of positive selection on the *KLK1* clade, in comparison with the other *KLK* paralogs (*SI Appendix*, Tables S5–S7). The branch-site-specific model identified 39 positively selected sites (pp ≥ 0.95) and computed a ω of 1.3 for this clade (*SI Appendix*, Table S5). Interestingly, 4 out of the 13 foreground branches that were identified to have undergone episodic positive selection (*P* ≤ 0.05) were *KLK1* genes identified from the genome of *S. paradoxus* (*SI Appendix*, Table S7). In combination, our findings suggest that at least 4 of the 8 solenodon *KLK1* genes (3 of the 7 *KLK1*s detected in venom) exhibit evidence of evolving under the influence of episodic positive selection. Thus, solenodon venom genes have evolved via the process of gene duplication coupled, in some cases, with episodic positive selection—a phenomenon that is consistent with the evolutionary histories of a number of toxin families found in other venomous animal lineages (1, 36).

Analysis of the genomic organization of kallikreins provides additional support for multiple independent origins of venom in eulipotyphlans. Mammalian kallikreins are found in a tandem array of linked genes, and while most exist as single-copy orthologs, *KLK1* and its upstream flanking gene *KLK15* show a more variable pattern (Fig. 4*B*). We found between 1 and 3 intact paralogs of these genes present in different mammals, and they are often interspersed by *KLK* pseudogenes or exon fragments (e.g., *Homo sapiens* has 3 *KLK1*-like genes, annotated as *KLK1*, *KLK2*, and *KLK3*, and 1 *KLK15* gene, with remnants of at least 1 other *KLK15*). Within Eulipotyphla, the representative mole (*Condylura cristata*), hedgehog (*Erinaceus europaeus*), and shrew (*Sorex araneus*) species analyzed, all of which are nonvenomous, were found to have 2 or 3 *KLK1* paralogs and a single *KLK15* gene, though the organization and orientation of these genes varied among species, suggesting independent evolutionary histories (Fig. 4*B*). Contrastingly, the solenodon genome revealed the presence of at least 8 *KLK15* and 8 *KLK1* paralogs, of which we detected 7 of the *KLK1*s proteomically in venom. While the contiguity of the solenodon genome is insufficient to perform synteny analysis, we note that multiple scaffolds contain *KLK1* and *KLK15* genes adjacent to one another (Fig. 4*B*), suggesting that the process giving rise to the extensive number of paralogs uniquely observed in this species may involve a duplication consisting of at least 1 of each of these genes. The combined findings from our molecular evolution and synteny analyses provide convincing evidence that solenodons have evolved multiple *KLK1* genes for use in their venom system, and that both solenodons and shrews have independently utilized *KLK1*s for a role in venom. Future work is required to assess whether *KLK1*s show similar evolutionary trajectories in other venomous eulipotyphlans (e.g., *Neomys* and *Crocidura* shrews), as comparative molecular data are currently unavailable for those species.

Next, we sought to infer the timing of the origin of venom in the order Eulipotyphla. To do so, we used ancestral trait reconstructions to reconstruct the character state for venom across this group. The resulting posterior probabilities (all >0.85) provided strong support for 4 independent origins of venom in this group: in solenodons, *Blarina* shrews, *Neomys* shrews, and *Crocidura* shrews (Fig. 4*C* and *SI Appendix*, Fig. S7). The unlikely alternative hypothesis of an early evolution of venom followed by the loss of this character state in multiple taxa required a single gain and at least 9 loss events, as *Blarina*, *Neomys*, and *Crocidura* are not closely related to one another within the Soricidae (last common ancestor ∼16 to 20 MYA; ref. 37) (Fig. 4*C* and *SI Appendix*, Fig. S7). While further research effort could change this interpretation—for example the future identification of additional shrew species or other eulipotyphlan families as venomous—the combination of diverse data types described above strongly suggest that both solenodons and shrews, which diverged from one another over 70 MYA (19), have independently evolved oral venom systems. Moreover, both these groups have independently recruited *KLK1*s for a role in venom, and thus provide a fascinating example of convergent molecular evolution. In this instance, molecular convergence seems likely to be underpinned by preadaptations, as both solenodons and shrews have evolved an oral venom system via the modification of submaxillary salivary glands (38, 39), and *KLK1* has previously been demonstrated to be an abundant component found in the salivary glands of a variety of mammals (*SI Appendix*, Fig. S8). Thus, *KLK1* likely existed as an abundant starting substrate in the oral secretions of ancestral eulipotyphlans before being independently selected for increased expression and diversification for use in the venom systems of multiple different eulipotyphlan groups. Therefore, solenodons and at least some shrews have achieved the same molecular solution for the composition of their venom, despite employing different morphological strategies to deliver those molecules (e.g., elaborate tooth grooves vs. rapid biting with pointed incisors and canines). While the venom delivery systems of many other venomous mammals (e.g., platypus and slow lorises) are distinct from the solely oral systems of eulipotyphlans, hematophagus vampire bats (e.g., *Desmodus rotundus*) also deliver venom produced in submaxillary glands via sharp incisors. Notably, *KLK1*-like proteins have previously been detected in their venom (40), alongside other serine proteases that activate plasminogen (41), thereby representing an intriguing example of molecular and functional venom convergence with their distant eulipotyphlan relatives (last common ancestor ∼87 MYA) (42).

Prior research has been unable to determine the ecological role of solenodon venom. It has previously been speculated that venom might facilitate prey capture, be a relictual trait, or be used for intraspecific competition or antipredator defense (8, 9, 17). The use of a hypotensive venom for defense would be unusual (although not unique) (43), as most defensive venoms cause acute pain to act as an immediate deterrent and to invoke learned avoidance behavior (44). However, solenodon bites inflicted on humans do not tend to result in such extensive pain, with inflammatory responses and secondary infections likely responsible for much of the resulting pathology (17, 45). Importantly, the insular Caribbean contained no native terrestrial mammalian predators before the mid-Holocene arrival of humans, who first introduced dogs, and then later cats and mongooses (46), suggesting that the evolution of a defensive venom is unlikely to be related to defense against predators. Although solenodons are known to be predated by owls and possibly other raptors (47), and coexisted prehistorically with giant Caribbean raptors that are now extinct (48), orally delivered venom seems unlikely to protect them from the talons of such avian predators.

There is also little evidence supporting the premise that solenodons use their venom for intraspecific purposes, such as for competition during breeding seasons (as in the platypus), or for resolving territorial disputes. Although some captivity case reports suggest that solenodons may have been killed following bites by other solenodon individuals (17), most captive accounts describe antagonistic encounters among solenodons being resolved without biting (49). Moreover, solenodons are relatively social animals; both species live in family groups comprising adults, subadults, and young, with multiple family groups of Cuban solenodons sharing the same den (45, 47, 50, 51). Although a lack of natural history reports documenting the behavior of these poorly known mammals limits our interpretation, we find no convincing evidence supporting the hypothesis for venom having evolved for an intraspecific purpose.

It appears most likely that the solenodon venom system evolved for capturing prey, in a manner analogous to, and in parallel with, venomous shrews. This hypothesis is supported by the convergent evolution of similar venom components (*KLK1s*) found in the solenodon and *Blarina* venom systems. However, *Blarina* shrews have a bipartite venom, consisting of both *KLK1*-like proteins that act on small vertebrates (11) and potent neuropeptides for the immobilization of invertebrates for long-term prey storage (52, 53). Although their feeding and hunting behavior is poorly understood, solenodons do not appear to “cache” their prey in this manner (49). Nonetheless, to test for the potential presence of neurotoxic venom activity, we assessed the activity of solenodon venom on nicotinic acetylcholine receptors (nAChRs) and voltage gated sodium channels (Na_v_), both of which are ion channels commonly targeted by venoms to cause immobilization via neuromuscular paralysis (1, 2). Solenodon venom exhibited no activity on either human muscle type or locust nAChRs at concentrations up to 50 µg/mL (Fig. 3*G*), but did display subtle, but significant, inhibitory activity at mammalian voltage-gated sodium channels (VGSCs) in whole-cell patch-clamp assays (*SI Appendix*, Fig. S9 and Table S8). While the observed reduction in peak current amplitude, as well as the inhibitory shifts in threshold for activation and inactivation of Na_V_ currents, could lead to paralysis, this was not apparent in in vivo toxicity studies using locusts (*Schistocerca gregaria*, *n* = 4) and centipedes (*Ethmostigmus rubripes*, *n* = 5), invertebrates representative of general groups that solenodons may consume in the wild (51). All animals survived 24 h after the intrathoracic injection of venom (up to 50 µg/g and 100 µg/g, respectively) with no signs of immobilization or incapacitation; it is therefore possible that the observed activity on Na_v_ channels may be specific to vertebrates. Irrespectively, we conclude from these data that solenodon venom is unlikely to function to paralyze invertebrates for prey storage, unlike that of *Blarina* shrews.

Kallikrein toxins present in *Blarina* shrew venom likely facilitate the capture of vertebrate prey, specifically by enabling relatively large-bodied prey to be subdued, which would otherwise be capable of escaping or defending themselves (11). To investigate the extent to which *S. paradoxus* feeds on vertebrate prey items, we assessed their dietary composition using a vertebrate DNA barcoding approach on 64 fecal samples collected during the dry and rainy seasons in Pedernales Province, Dominican Republic. Our conservative “frequency of occurrence” approach detected evidence of vertebrate prey in 12.3% of the fecal samples analyzed (Fig. 1), demonstrating that while solenodons feed predominately on invertebrates (54), vertebrate prey make up a considerable proportion of their diet. These findings are in line with a number of natural history reports describing solenodons feeding on amphibians, reptiles, and occasionally nestlings and ground-dwelling birds (45, 47, 50, 51). Although many vertebrate species remain as potential solenodon prey items in Cuba and Hispaniola, it is also possible that the solenodon venom system initially evolved for capturing vertebrate prey, and is now at least partly relictual due to the extinction of considerable amounts of regionally endemic vertebrate biodiversity (e.g., lizards, birds, and mammals) from their habitats. In particular, all of the smallest-bodied native Caribbean land mammals (nesophontids, spiny rats, small hutias), which might have ancestrally constituted solenodon prey species, became extinct following European arrival in the insular Caribbean ∼500 y ago (46). Indeed, our sample site exhibits considerable evidence of anthropogenic impacts (e.g., mosaic agriculture-forest habitat), and thus the diet reported here may heavily reflect flexible responses to anthropogenic resources and the prey types currently available to solenodons. Based on venom composition, dietary data, and limited available natural history reports, we conclude that the solenodon venom system likely evolved for overpowering and subduing occasional (and perhaps previously more abundant) vertebrate prey items.

Our findings collectively highlight the unique significance of eulipotyphlans within mammals for having multiple origins of venom and venom delivery systems. Venom has evolved more times in this group than in all other mammals combined, and on more instances than those found within any other class of vertebrates, other than bony fish. The reason for the frequency of venom evolution in eulipotyphlans remains unclear, but considering the majority of these venom origins relate to shrews, a group of animals well known for having high metabolic rates that require frequent feeding (10), venom may be a valuable adaptation that facilitates their near-continual foraging lifestyle. In solenodons, venom also appears to facilitate prey capture, but additional work is required to fully elucidate the nature of this venom, such as 1) compiling extensive natural history observations of foraging behavior; 2) testing solenodon venom on natural prey items; 3) comparisons of venom composition and function between the 2 solenodon species, and between male and female individuals; and 4) further investigation of solenodon dental and mandibular morphology to understand their prey handling capability. Nonetheless, our findings highlight the evolutionary novelty of the solenodon venom system and stress the importance of studying and conserving endangered species in order to protect both ecological diversity and their utilitarian value, which in this case is most relevant when considering the bioactive compounds found in their toxic secretions (7, 55). Ultimately, our work reveals a surprising case of convergent molecular evolution, whereby *KLK1*s have been independently recruited for use in the nonhomologous venom systems of shrews and solenodons. These findings highlight that the molecular constituents of eulipotyphlan venom systems may be subjected to constraints that limit the options available for the evolution of venom, as these lineages have highly divergent phylogenetic backgrounds and different morphological adaptations for delivering these molecules. Our findings therefore emphasize that distinct structural phenotypes, encapsulated by variation in venom delivery systems, can yield equivalent functions, and more generally, they reinforce the broad value of studying natural toxin systems to elucidate fundamental evolutionary processes.

## Materials and Methods

Detailed materials and methods can be found in *SI Appendix*.

### Genomics.

The *S. paradoxus* genome was constructed using high molecular weight DNA isolated from the blood of a captive male adult individual. Paired-end library sequencing was performed on an Illumina HiSeq 2500 instrument with 250-bp reads. The assembly was undertaken using DISCOVAR de novo (21), before annotation with MAKER (24), with Augustus (56) implemented to facilitate gene prediction. We then used BUSCO (22) to individually assess the quality of the genome assembly and annotation.

### Proteomics.

Venom and saliva samples were collected from 2 wild-caught adult male Hispaniolan solenodons. We used reduced SDS-PAGE gel electrophoresis for initial visualization of venom and saliva proteins (10 µg). Shotgun proteomics was performed by digesting 5 µg of each sample with trypsin, before analysis by LC-MS/MS. Decomplexed bottom-up proteomics were performed as previously described (27). Samples (1 mg) were separated via reverse-phase HPLC, then reduced and analyzed by SDS-PAGE, and protein bands subjected to in-gel tryptic digestion and analyzed by LC-MS/MS using an Orbitrap XL (Agilent, Germany). For top-down proteomics we used 0.2 mg of venom and saliva for reduced and nonreduced HPLC high-resolution (HR) MS/MS measurements. Top-down LC-electrospray ionization-HR-MS experiments were performed on an LTQ Orbitrap XL (Agilent) in data-dependent acquisition mode. For all proteomic experiments, resulting MS2 spectra (57) were matched against translations of the protein-encoding genes predicted from the genome. Full details are displayed in *SI Appendix*, File S1.

### Evolutionary Analyses.

The 26 *KLK* genes identified in the solenodon genome were used as queries for BLAST searching the National Center for Biotechnology Information nonredundant and Ensembl tetrapod genome databases. Resulting nucleotide and amino acid sequences were aligned (*SI Appendix*, Files S2 and S3) and subjected to Bayesian inference (2 × 10^8^ generations, parallel runs = 4, Markov chain Monte Carlo [MCMC] simulations = 6) and the nucleotides also to maximum likelihood (subtree pruning and regrafting method, 100 bootstrapping replicates) analyses. To investigate the influence of selection on tetrapod *KLK*s we employed 1) site-, branch-, and branch-site maximum likelihood models implemented in CodeML of the PAML package (35); 2) MEME analyses (58); 3) FUBAR analyses (59); and 4) the adaptive branch-site random effects likelihood (aBSREL) approach (60). Three-dimensional homology models were generated for the various tetrapod *KLK*s using the Phyre2 server (61), and PyMOL (PyMOL Molecular Graphics System, Schrödinger, LLC) was used for visualization. Analyses of hydropathicity and charge of *KLK1* regulatory loops (34) were calculated using the ProtParam tool of the ExPASy Bioinformatics Resource Portal, with statistical comparisons performed using unpaired 2-tailed *t* tests in GraphPad Prism (La Jolla, CA). De novo annotation of *KLK* exons and synteny comparisons of mammalian genomic data were undertaken as recently described (62). Ancestral trait reconstructions were performed with Ape (63) and Phytools (64) in R, and the marginal ancestral states (empirical Bayesian posterior probabilities) were estimated for each node in a eulipotyphlan species tree derived from prior studies (19, 37). A stochastic character mapping analysis (65) was performed for 1,000 simulations, and a trait density map was generated to depict the posterior probabilities of states across the tree.

### In Vitro Venom Function.

Degradation gel electrophoresis experiments were performed using 5 µg of substrate (HMW kininogen or fibrinogen) and 5 µg of venom or saliva. Samples were either incubated at 37 °C for 60 min or loaded directly onto SDS-PAGE gels for electrophoretic separation under reducing conditions. For serine protease activity, we used a chromogenic assay (*n* = 3 independent repeats) with the specific substrate S-2288 (Cambridge Biosciences). Samples (1 µg) were plated in triplicate into 384-well plates, overlaid with Tris buffer (100 mM Tris, 100 mM NaCl, pH 8.5), and 6 mM of S-2288, and absorbances were measured at 405 nm kinetically. Control (PBS) readings were subtracted and the rate of substrate consumption was calculated by measuring the slope between 0 and 5 min. To monitor plasminogen cleavage we used a modified kinetic assay (*n* = 3 independent repeats) (66) to detect the resulting plasmin activity via cleavage of the H-D-Val-Leu-Lys-AMC fluorescent substrate (I-1390, Bachem). One microgram of venom/saliva was prepared in 10 µL of assay buffer (100 mM Tris⋅HCl pH 7.5, 0.1% BSA), plated in triplicate into 384-well plates, and overlaid with 50 µL/well of 200 ng/mL plasminogen (Sigma-Aldrich) and 5 µM of substrate. Fluorescence was measured kinetically for 45 min (excitation 355 nm, emission 460 nm) and areas under the curves were calculated for the 0- to 30-min interval. Statistical analyses (unpaired 2-tailed *t* tests) for the kinetic assays were performed in GraphPad Prism. To identify the toxins responsible for plasminogen activating activity, we followed the previously described approach (66), whereby venom (250 µg) was fractionated by LC in parallel with at-line nanofractionation and subsequent identification of bioactives from the plasmin assay identified via nanoLC-MS/MS analysis of tryptic digests of the corresponding fractions. Details of the resulting peptide spectrum matching are displayed in *SI Appendix*, File S4. For patch-clamp electrophysiology experiments, we used: 1) TE671 human rhabdomyosarcoma cells endogenously expressing embryonic muscle-type nAChRs and Na_v_1.7 VGSCs and 2) locust (*S. gregaria*) primary neurons (natively expressing insect neuronal nAChRs), the latter of which were dissected from the mushroom bodies of sixth instar locusts. Patch pipettes (resistance 5 to 7 MΩ) were filled with a caesium pipette solution (140 mM CsCl, 10 mM NaCl 1 mM MgCl_2_, 11 mM EGTA and 5 mM Hepes, pH 7.2 with CsOH). The bath solution for TE671 cells was 135 mM NaCl, 5.4 mM KCl, 1 mM CaCl_2_, 1 mM MgCl_2_, 5 mM Hepes and 10 mM d-glucose (pH 7.4 with NaOH), and for locust neurons, 180 mM NaCl, 10 mM KCl, 2 mM CaCl_2_, 10 mM Hepes, pH 7.2. Whole-cell currents were monitored using an Axopatch 200A (Axon Instruments) patch-clamp amplifier and venom and agonist were applied to cells using a DAD-12 Superfusion system (Adams and List Associates). The series resistance was compensated by 75% to minimize any voltage errors, and data were filtered at 10 kHz.

### In Vivo Venom Function.

Groups of fifth instar desert locusts (*S. gregaria*, *n* = 4) and juvenile giant centipedes (*E. rubripes*, *n* = 5) were injected intrathoracically with various solenodon venom doses (0.1 to 100 µg/g) alongside controls (insect Ringer’s saline) and their status (alive, incapacitated, dead) was monitored for at least 24 h. Comparisons of the physiological responses of mice (20 g, male CD1, Charles River) dosed i.v. with solenodon venom (25 µg/g, *n* = 3) and PBS (control, pH 7.2, *n* = 3) were undertaken using a MouseOx pulse-oximeter monitoring system (MouseOx, Harvard Apparatus). Measurements were collected at 5 different times for each animal (baseline, 1 min, 15 min, 30 min, and 45 min postadministration). We examined the effect of solenodon venom on the blood pressure of anesthetized rats (male Sprague-Dawley, 250 to 320 g) by connecting a carotid artery cannula to a PowerLab/400 system via a Gould Statham P23 pressure transducer. Blood pressure was allowed to stabilize for at least 10 min before venom (1 mg/kg; *n* = 5) was administered through the jugular vein and flushed with saline (0.2 mL).

### Dietary Analyses.

DNA was isolated from 64 samples collected opportunistically from fresh *S. paradoxus* fecal samples (Pedernales Province, Dominican Republic; 40 in the dry season; 24 in the wet season) and probed for the presence of vertebrate prey DNA using primers specific to 12S and 16S ribosomal genes. Resulting DNA was sequenced on an Ilumina MiSeq instrument and operational taxonomic units were identified via BLAST searches of the GenBank nonredundant nucleotide and the Barcode of Life Data Systems (BOLD) databases, using 85% identity thresholds. To calculate the frequency of occurrence of vertebrate prey, we summed the presence of each identified food item across all 64 samples and divided that figure by the total number of fecal samples.

### Permissions.

Ethical permission, collection permits, and export permits for solenodon samples were granted by ZOODOM and the Ministerio de Medio Ambiente y Recursos Naturales (no. 2577 and VAPB-02368). The murine in vivo study was conducted using protocols approved by the animal welfare and ethical review boards of the Liverpool School of Tropical Medicine and the University of Liverpool and performed under licensed approval of the UK Home Office, in accordance with the Animal [Scientific Procedures] Act 1986 (UK) and institutional guidance on animal care. The rat in vivo study was approved by the Monash Animal Research platform (MARP) Animal Ethics Committee, Monash University, Australia (MARP/2017/147).

## Supplementary Material

Supplementary File

Supplementary File

Supplementary File

Supplementary File

Supplementary File
