# Supplementary material for: Solenodon genome reveals convergent evolution of venom in eulipotyphlan mammals
Source: Proc Natl Acad Sci U S A. 2019 Nov 26;116(51):25745–55. doi: 10.1073/pnas.1906117116 (PMC6926037; doi:10.1073/pnas.1906117116)
Supplement: Supplementary File [file pnas.1906117116.sapp.pdf]

PNAS
www.pnas.org

Supplementary Information for

Solenodon genome reveals convergent evolution of venom in eulipotyphlan mammals

Nicholas R. Casewell, Daniel Petras, Daren C. Card, Vivek Suranse, Alexis M. Mychajliw, David Richards, Ivan Koludarov, Laura-Oana Albulescu, Julien Slagboom, Benjamin-Florian Hempel, Neville M. Ngum, Rosalind J. Kennerley, Jorge L. Brocca, Gareth Whiteley, Robert A. Harrison, Fiona M. S. Bolton, Jordan Debono, Freek J. Vonk, Jessica Alföldi, Jeremy Johnson, Elinor K. Karlsson, Kerstin Lindblad-Toh, Ian R. Mellor, Roderich D. Süssmuth, Bryan G. Fry, Sanjaya Kuruppu, Wayne C. Hodgson, Jeroen Kool, Todd A. Castoe, Ian Barnes, Kartik Sunagar, Eivind A. B. Undheim and Samuel T. Turvey.

Nicholas R. Casewell
Email: nicholas.casewell@lstmed.ac.uk

This PDF file includes:

SI Methods
Figures S1 to S9
Tables S1 to S8
SI References

SI Appendix Methods

Sample collection

Solenodon blood samples for genomics were obtained via tail vein venepuncture from a single male adult Hispaniolan solenodon from the northern Dominican Republic (*Solenodon paradoxus paradoxus*) housed off exhibit at the Dominican Republic National Zoo, ZOODOM. Immediately after extraction, the blood was flash frozen in liquid nitrogen, and then stored cryogenically until DNA extraction. Venom and saliva samples were collected from two male, adult, wild-caught Hispaniolan solenodons from the southern Dominican Republic (*S. p. woodi*) in the Las Mercedes area of Pedernales Province (~18°05' N, 71°64' W). The animals were caught when foraging at night, sampled *in situ* and then immediately released. Saliva was collected first by manually pipetting from the posterior of the mouth prior to venom stimulation. For venom extraction, the animals were carefully restrained and encouraged to bite on soft rubber tubing, which stimulated the secretion of venom, which was collected into a falcon tube and then transferred immediately to cryovials for flash freezing in liquid nitrogen. Following cryogenic transportation to the UK, both venom and saliva samples were lyophilised and stored long-term at 4°C. Of the saliva samples collected, one was discarded due to insufficient protein remaining to perform downstream analyses. Ethical permission, collection permits and export permits were granted by the Dominican Republic National Zoo (ZOODOM) and the Ministerio de Medio Ambiente y Recursos Naturales (#2577 and #VAPB-02368).

Genomics

Genome sequencing and assembly. We isolated high molecular weight DNA from 500 µl of *S. paradoxus* blood using the QIAamp DNA blood midi kit (Qiagen) as per manufacturer's instructions. The integrity of the resulting DNA was analysed on an Agilent 2200 TapeStation with an Agilent High Sensitivity D1000 ScreenTape System, and the DNA concentration was determined using the Invitrogen Qubit dsDNA HS assay kit. The samples (1-3 µg) were then fragmented on a Covaris E220 Instrument using the 400 bp standard program (10% Duty cycle, 140 PIP, 200 cycles per burst, 55s). Fragmented samples then underwent SPRI double size selection (0.55X, 0.7X), followed by PCR-free Illumina library construction according to the manufacturer's instructions (KAPA LTP Library Preparation Kit, #KK8232), and employing PCR-free adapters from Illumina (#FC-121-3001). The final library fragment size distribution was then determined on an Agilent 2100 Bioanalyzer with High Sensitivity DNA Chips. We sequenced a single paired-end library on a single-lane of the Illumina HiSeq2500 set for Version 2 chemistry and with 250 bp reads. We then applied Discovar de novo (https://software.broadinstitute.org/software/discovar/blog/) assembly to the 2x250 bp read groups using the following commands:

```
DiscovarDeNovo READS=[READFILE] /
OUT_DIR=[SPECIES_ID]//[SPECIES_ID].discovar_files /
NUM_THREADS=24 MAX_MEM_GB=200G
```

Assembly assessment. In order to assess the quality of our genome assembly and compare it to the recently-published assembly generated by Grigorev et al. (1), we calculated contig N50, scaffold N50, and percent Ns (i.e., gaps). While scaffold N50 and percent Ns were qualitatively similar, the contig N50 for our assembly was approximately four times greater than in Grigorev et al. (1) (SI Appendix Table S1). We also ran BUSCO (2) in 'genome' mode with default parameters to assess genome assembly quality under the assumption that better assemblies contain a higher percentage of complete BUSCOs. Of 4,104 total mammalian BUSCOs searched, we found that 97.6% were identified as either complete (92.9%) or partial (4.7%) in our annotation protein set. The substantially greater number of complete BUSCOs identified in our assembly versus that of Grigorev et al. (1) (SI Appendix Table S1) indicates a superior genome assembly.

Genome Annotation. First, we used BLASTN (BLAST v. 2.2.28+; (3)) and an available reference whole mitochondrial genome (NCBI accession KU697362.1; length 16,455 bp; (4)) to identify the mitochondrial scaffold from our *de novo* genome assembly. The mitochondrial sequence was largely matched in two large BLAST hits to a single assembled 16,556 bp scaffold, which stood out against other off-target matches. We used the MITOS WebServer (5) to annotate all protein-coding, tRNA, and rRNA genes.

Next, we used RepeatModeler v. 1.0.4 (http://www.repeatmasker.org) with default settings to identify repeats specific to the *Solenodon paradoxus* genome. We split the resulting library into known and unknown elements and further annotated unknown elements using a TBLASTX search against the known repetitive elements and Tetrapoda repeats from the RepBase library (release 20170807 (6)). Approximately one-third of the unknown elements were classified in this manner, and the final *S. paradoxus* repeat library contained 41 remaining unknown elements and a total of 337 elements.

MAKER v. 2.31.9 (7–9) was used to produce a complete annotation of the *S. paradoxus* genome. To annotate repetitive content within MAKER, we set the 'model_org' setting to 'tetrapoda' to leverage curated

Tetrapoda repeats from RepBase (release 20150807) and we provided the additional classified *Solenodon* repeat library containing species-specific consensus sequences. Overall, 29.4% of the genome sequence was identified as repetitive, with 2.8% of repetitive content classified as simple repeats and 26.6% as complex repeats.

To annotate protein-coding regions, we provided the amino acid sequences for the complete gene sets of three closely related species with whole-genome annotations already available: star-nosed mole (*Condylura cristata* ConCri1.0) European shrew (*Sorex araneus* SolAra2.0), and western European hedgehog (*Erinaceus europaeus* EriEur2.0). Genome annotations for these three species were obtained from NCBI and gene models were based on homology with existing transcript and protein sequence data from RefSeq and GenBank for taxonomically-relevant species, and appropriate RNA-Seq data available through the SRA database for the target organism (existing RNA-seq data were confirmed for *Condylura cristata* and *Erinaceus europaeus*). Given that *S. paradoxus* is known to be venomous, we also supplied amino acid sequences for genes we identified as venom orthologs from other mammal species. The 'max_dna_length' setting was increased to 300,000 and the 'split_hit' setting was increased to 20,000. The 'protein2genome' option was turned on to allow MAKER to use the results of the protein alignment to create gene models. All other settings were left in default state.

Following the first round of MAKER, we used the MAKER gene models to train two *ab initio* gene prediction programs: SNAP v. 2006-07-28 (10) and Augustus v. 3.2.3 (11). SNAP was run with default settings on all resulting gene models. Augustus was trained within the BUSCO pipeline (2) (i.e., using the '--long' setting) by providing the sequences for coding regions (full annotated transcripts plus 1000 bp up- and downstream) and using the 'genome' mode and 4,104 mammalian BUSCOs.

The trained SNAP and Augustus HMM models were fed into a second MAKER run, using the same settings in all cases except that `protein2genome` was turned off so that MAKER would use the gene models produced by SNAP and Augustus, informed by hints from the empirical data, to produce the final gene models. We evaluated the gene models by examining distributions of Annotation Edit Distance (AED; (12)) scores and gene model numbers, lengths, and numbers of exons. JBrowse (13, 14) was used to visualize all gene models and the evidence from protein alignments. We found that Augustus was producing gene models that were in line with empirical evidence from protein alignments, while SNAP produced more spurious and less informative gene models, and as such, we used only Augustus predictions for subsequent rounds of MAKER. We repeated the gene predictor training and MAKER run, as described an additional time until gene models were stable and well supported by empirical data.

The resulting gene annotation contained 18,112 protein-coding genes. 95.8% of gene models had an AED at or below 0.5, indicating a high-quality annotation well supported by empirical protein alignments. Moreover, we ran BUSCO as above, but in 'protein' mode using the final Augustus HMM model used for annotation (see above), to quantify the number of mammalian BUSCOs (N = 4,104 total) across annotated proteins. We found that 95.7% of BUSCOs were identified as either complete (84.8%) or partial (10.9%) in our annotation protein set. The moderate recovery of complete BUSCO genes from proteins alone suggests that the evolutionary distance between *S. paradoxus* and the other mammal protein sets used for annotation, or the overall protein representation contained within those protein sets, may have impacted upon the final protein models.

Lastly, to better understand the identity of annotated genes, we used the protein sequences to annotate gene models based on homology to several outside sources. We used InterProScan v. 5.27-66.0 (15) to match proteins against the InterPro database version 66.0 (16). A total of 15,782 genes were annotated based on the PFAM database (17) and we were able to ascribe PANTHER gene ontology IDs (18) to 17,595 genes. We also performed both reciprocal best BLASTP and stringent unidirectional BLASTP searches using a custom script (orthorbb v. 2.1; https://github.com/darencard/ContigAnnotator/blob/master/orthorbb) between the protein annotations for *Solenodon* and (1) combined annotated proteins from star-nosed mole, European shrew, and western European hedgehog, and (2) the entire UniProt/SwissProt release 2017-11-22 (19). We used e-value cutoffs of 1e-5 for the reciprocal best BLASTP searches and 1e-8 for the stringent unidirectional BLASTP searches. When summarizing these searches, we prioritized the results of the reciprocal best BLASTP over the one-way BLASTP, as these reflect higher confidence in homology between *Solenodon* proteins and proteins of the other species. 18,022 proteins were matched to homologs in the other three mammal species (15,034 based on reciprocal best BLASTP and 2,988 based on one-way BLASTP). Matching homologs for 17,937 genes were identified based on reciprocal best BLASTP (14,688) and one-way BLASTP (3,249) analyses using the Uniprot database.

Data repository. The resulting genome assembly for *Solenodon paradoxus* has been deposited to the Genome database of NCBI under accession number RJWH00000000, and linked to the BioProject identifier PRJNA399371 and the BioSample identifier SAMN07678062. A dedicated website for the *Solenodon paradoxus* assembly and genome annotation has also been established (https://darencard.github.io/solPar). Repeat and gene annotation files have also been deposited to Figshare

(https://doi.org/10.6084/m9.figshare.7640456). We have also created a Genome Hub that can be accessed through the UCSC Genome Browser via the following link (https://genome.ucsc.edu/cgi-bin/hgHubConnect), followed by clicking on the “My Hubs” tab and then copying the following URL (https://de.cyverse.org/anon-files/iplant/home/darencard/solPar1_hub/assembly_hub.hub.txt) and clicking “Add Hub”.

Proteomics

SDS-PAGE gel electrophoresis. We first visualised the protein composition of the two venom samples and the saliva sample by one-dimensional SDS-PAGE gel electrophoresis. Ten micrograms of each sample (1 mg/ml) were added to reducing protein loading buffer at a ratio of 1:1 and incubated at 100°C for ten minutes. The samples were then loaded onto ten-well Mini-PROTEAN TGX precast AnykD gels (Bio-Rad), alongside 5 µl of protein marker (Broad Range Molecular Marker, Promega), and run at 100 V for 60 mins using a Mini-PROTEAN Tetra system (Bio-Rad). The gel was then stained with Bio-Safe Coomassie Stain (Bio-Rad) and destained with water for visualisation.

Bottom-up proteomics. Bottom-up analyses were carried out on either crude or decomplexed venom and saliva from the Hispaniolan solenodon (*S. paradoxus*). For our shotgun experiments, we digested 5 µg venom and saliva with trypsin (sequence grade, Sigma Aldrich) and analysed by LC-MS/MS. Briefly, samples were dried and re-dissolved in 4 M urea, 10% acetonitrile (ACN), 100 mM ammonium bicarbonate, pH 8. Cysteines were then reduced by incubating with 5 mM dithiothreitol (DTT) at 70 °C for 5 min and alkylated with 10 mM iodoacetamide at 37 °C for 90 min. The reduced and alkylated samples were then digested by incubating with 30 ng/µl trypsin overnight at 37 °C in 2 M urea, 10% ACN, 100 mM ammonium bicarbonate, pH 8, at a final substrate to enzyme ratio of approximately 100:1. The digested samples were desalted using a C18 ZipTip (ThermoFisher, Waltham, MA, USA), dried using vacuum centrifugation, dissolved in 0.5% formic acid (HFo) and 2 µg analysed on an AB Sciex 5600 TripleTOF (AB SCIEX, Framingham, MA, USA) equipped with a Turbo-V source heated to 550 °C. Tryptic peptides were fractionated on a Shimadzu (Kyoto, Japan) Nexera UHPLC with an Agilent Zorbax stable-bond C18 column (Agilent, Santa Clara, CA, USA) (2.1 x 100 mm, 1.8 µm particle size, 300 Å pore size), using a flow rate of 180 µl/min and a gradient of 1–40% solvent B (90% ACN, 0.1% HFo) in 0.1% HFo over 60 min. MS1 spectra were acquired at 300–1800 m/z with an accumulation time of 250 ms and selecting the 20 most intense ions for MS2 scans acquired at 80–1400 m/z with an accumulation time of 100 ms and optimized for high resolution. Precursor ions with a charge of +2 to +5 and an intensity of at least 120 counts/s were selected, with a unit mass precursor ion inclusion window of ±0.7 Da and excluding isotopes within ±2 Da for MS/MS. To identify venom and salivary proteins, we used Protein Pilot v5.0 (AB SCIEX, Framingham, MA, USA) to search the MS/MS spectra against all translated predicted protein encoding genes in the *S. paradoxus* genome, allowing for both biological modifications and amino acid substitutions. False positives were identified using decoy-based false discovery rates (FDR) as estimated by Protein Pilot, and only protein identifications with a corresponding local FDR of <0.5% were considered significant. Genome matches were then annotated in Blast2GO (20) and gene ontology (GO) terms ascribed.

Analysis of decomplexed venom was done according to (21). In short, 1 mg of crude *S. paradoxus* venom was dissolved in aqueous solution, including 1% HFo and 5% ACN, to a final concentration of 10 mg/ml. Insoluble material was removed by centrifugation at 20,000 x *g* for 5 min. Dissolved venom was injected to an Agilent 1260 semi-preparative reverse-phase (RP) HPLC system (Agilent, Waldbronn, Germany) coupled to a Supelco Discovery Biowide C18 column (300 Å pore diameter, 4.6 x 150 mm column size, 3 mm particle size). The venom components were eluted with a linear gradient of 0.1% HFo in water (solution A) and 0.1% HFo in ACN (solution B) with a flow rate set to 1 mL/min. The gradient started isocratically (5% B) for 5 min, followed by linear gradients of 5-40% B for 95 min, 40-70% for 20 min, 70% B for 10 min, and finally end with a re-equilibration at 5% B for 10 min. Peak detection was performed by means of UV detection at l = 214 nm using a diode array detector (DAD). The peak fractions were collected manually and dried overnight in a vacuum centrifuge. The fractions containing peptides, previously determined by intact mass profiling, were re-dissolved in 20 µL of 5% ACN containing 0.1% HFo and directly submitted to LC-MS/MS analysis. The protein-containing fractions were then chemically reduced with DTT, subsequently heated at 90 °C for 10 min and separated via SDS-PAGE (15% polyacrylamide gels). Thereafter coomassie-stained bands were excised from the gel and subjected to in-gel digestion. In the first step bands were treated with a reduction solution (10 mM DTT in 25 mM $(NH_4)HCO_3$, pH 8.3, for 30 min at 65 °C), then a alkylation step (50 mM iodoacetamide in 50 mM $(NH_4)HCO_3$, pH 8.3, for 30 min at 25 °C in the dark) was applied, before in-gel trypsin digestion (12 h at 37 °C with 66 ng sequencing-grade trypsin/mL in 25 mM $(NH_4)HCO_3$, 10% ACN; 0.25 mg/sample). Samples were dried in a vacuum centrifuge, tryptic peptides were re-dissolved in 20 µl of 5% ACN containing 0.1% HFo and subsequent submitted to LC-MS/MS analysis using an Orbitrap XL hybrid mass spectrometer (Thermo, Bremen, Germany) coupled with an Agilent 1260 HPLC system (Agilent, Waldbronn, Germany), using a flow rate of 0.3 ml/min. The HPLC system was connected to a Grace Vydac 218MSC18 column (2.1 x 15 mm, 5 mm). A gradient was applied using 0.1% HFo in water (solution A) and ACN (solution B) and started isocratically with 5% B for 2 min, followed by an increase over 10 min from 5 to

40% B, then 40-99% B over 15 min, 99% B was held for 5 min with a final re-equilibration phase at 5% B for 5 min. MS experiments were performed on an Orbitrap analyzer with R = 15,000 at *m/z* 400 and maximum filling time of 200 ms for both survey and first product ion scans. MS/MS fragmentation of the most intense ion was performed in the LTQ using CID (30 ms activation time); the collision energy was set to 35%. Precursor-ion isolation was performed within a mass window of *m/z* 2. Dynamic exclusion was set up for a mass window of *m/z* 3 for up to 50 precursor ions with a repeat of 2 within 30 s. (22) MS2 spectra were searched against all translated predicted protein encoding genes in the *S. paradoxus* genome, and a set of protein typical contaminant (common Repository of Adventitous Proteins; CRAP), in total 18,272 sequences. Mass accuracy of XTandem! was set to 10 ppm for precursor mass and 0.2 m/z for MS2 level. Alkylation of Cys was set as fixed modification and acetylation of N-term, Lys as well as oxidation of Met were allowed as variable modifications. FDR was estimated through target-decoy approach and a cut-off of 0.01 was applied. All PSMs were validated manually and only protein IDs with at least two PSMs were considered.

Top-down proteomics. For top-down proteomics, we dissolved 0.2 mg crude venom and saliva in 20 ul aqueous 1% (v/v) HFo, to a final concentration of 10 mg/ml, and centrifuged at 20,000 x *g* for 5 min. Dissolved samples were then mixed with 60 µl of citrate buffer (0.1 M, pH 4.2) and divided into 40 µl each. One sample was mixed with 10 µl ultra-pure water, while the other sample was mixed with 10 µl of *tris*(2-carboxyethyl)-phosphine (TCEP, 0.5 M) to chemically reduce existing disulfide bonds. The reaction mixtures were incubated for 30 min at 65 °C. Subsequently, the samples were centrifuged at 20,000 x *g* for 5 min, before 20 µl of both reduced and non-reduced samples were submitted to HPLC-high-resolution (HR) MS/MS measurements. Top-down LC-ESI-HR-MS experiments were performed on an LTQ Orbitrap XL mass spectrometer (Thermo, Bremen, Germany) coupled to an Agilent 1260 HPLC system (Agilent, Waldbronn, Germany), using a Supelco Discovery 300 Å C18 (2 x 150 mm, 3 mm particle size) column. The flow rate was set to 0.3 ml/min and a gradient of 0.1% HFo in water (solution A) and 0.1% HFo in ACN (solution B) was used. The gradient started isocratically (5% B) for 5 min, followed by an increase from 5 to 40% B over 85 min, 40-70% over 20 min, thereafter a washout at 70% B for 10 min and ended in a re-equilibration phase at 5% B for 10 min. ESI settings were 7 L/min sheath gas; 25 L/min auxiliary gas; spray voltage, 4.8 kV; capillary voltage, 30 V; tube lens voltage, 170 V and capillary temperature, 330 ºC. The survey scan was performed with mass resolution (R) of 100,000 (at *m/z* 400). The MS2 spectra were obtained in a data-dependent acquisition (DDA) mode with R = 100,000 (at m/z 400) and two scan events, where the most abundant ion of the survey scan with calculable charge was selected. The FTMS measurements were performed with 2 micro scans and 500 ms maximal fill time. AGC targets were set to 10_6 for full MS scans and $3x10_5$ for MS/MS scans. The normalized collision energy (CID) was adjusted to 30% and the high energy C-trap dissociation (HCD) to 35%. The activation time was set to 30 msec and the default charge state to z = 10 for the CID scan event or z = 7 for the HCD scan event. The precursor selection window was set to 2 m/z. Dynamic exclusion was performed with a 3 m/z exclusion window for precursor ions with 2 repeats within 10 s. The exclusion list contained maximal 50 ions for a duration of 20 s.

For data analysis, raw data were converted to .mzXML files using MSconvert of the ProteoWizard package (version 3.065.85) and multiple charged spectra were deconvoluted using MS-Deconv (version 0.8.0.7370). The maximum charge was set to 30, maximum mass was set to 50,000, signal-to-noise threshold was set to 2, and m/z tolerance was set to 0.02 amu. Protein spectra matching was performed using TopPIC version 1.0.0 (http://proteomics.informatics.iupui.edu/software/toppic/) against all translated predicted protein encoding genes in the *S. paradoxus* genome, and a set of protein sequences found as typical contaminants from the common Repository of Adventitious Proteins (CRAP), in total 18,272 sequences. TopPIC mass error tolerance was set to 20 ppm. A false discovery rate (FDR) cut-off was set to 0.01. Maximal allowed unexpected PTMs was set to one. The deconvolution of isotopically resolved spectra was carried out by using the XTRACT algorithm of Xcalibur Qual Browser (Thermo, Bremen, Germany).

The intact mass extracted ion chromatograms (XICs) were generated by deconvolution of the MS raw data using XTRACT of the Xcalibur Qual Browser version 2.2 (Thermo, Bremen, Germany). MZmine2 (version 2.25) was used for intact mass feature findings of the mono-isotopic deconvoluted MS spectra for the native venom and saliva samples. The mass alignment for the creation of XICs was performed with a minimum peak width of 30 s and 3.0e4 peak height. A 1.0e4 signal intensity threshold for the peak selection was used and mass error tolerance was set to 10 ppm. The baseline cutoff algorithm for chromatographic deconvolution was set to 1.0e4 signal threshold and the maximum peak width was set to 10 min. Feature alignment was performed with 10 ppm mass accuracy and 0.5 min retention time tolerance.

Details of all peptide/protein spectrum matching for the various proteomic experiments are displayed in SI Appendix File S1.

Data repository. Mass spectrometry based proteomics data (.mgf, .raw, .mzXML and PSM/ PrSM output files as well as deconvoluted spectra) have been deposited to ProteomeXchange

(http://proteomecentral.proteomexchange.org) with the ID PXD009593 via the Mass Spectrometry Interactive Virtual Environment (MassIVE, https://massive.ucsd.edu/) with the accession number MSV000082307.

Evolutionary analyses

Phylogenetic analyses. To infer the molecular evolution of tetrapod kallikreins, we used the 26 nucleotide sequences identified in the *Solenodon paradoxus* genome as queries, and retrieved homologous sequences for representative vertebrates, with a focus on mammals, from NCBI's 'non-redundant' database (http://www.ncbi.nlm.nih.gov/) and vertebrate genomes in Ensembl (23). This dataset was supplemented with sequences sourced from a prior phylogenetic analysis of the kallikrein gene family (24). The resulting nucleotide sequences were aligned using MUSCLE (25), and are displayed in SI Appendix File S2. Next, Bayesian inference was implemented in MrBayes v3.2.6 (26) for phylogenetic reconstructions. The analysis was executed for $2x10_8$ generations with four parallel runs each with six simultaneous Markov chain Monte Carlo simulations, sampling every 100_{th} tree and parameter set, and using a mixed model of evolution. The log-likelihood score of every sample was plotted against the number of generations to attain the point where the log-likelihood scores asymptote. The first 25% of the sampled trees and model parameters were discarded as burn-in, while the remaining were used for generating the consensus tree. The posterior probabilities for the nodes were estimated by creating a majority-rule consensus tree from all trees selected after burn-in. Phylogenetic reconstructions were also performed using a maximum likelihood approach implemented in PhyML 3.0 (27). The optimal model of nucleotide substitution was identified as GTR+G+I, and the tree topology was determined by implementing this model and using the Subtree Pruning and Regrafting (SPR) method, with 100 bootstrapping replicates. For amino acid analyses, we used a pruned dataset that retained representative sequences from *Homo*, *Mus* and *Solenodon* for all kallikrein paralogs (and other more distantly related serine proteases), in addition to a broad range of *KLK1* sequences sourced from a diverse array of mammalian taxa. The resulting dataset was aligned using MUSCLE (25), and the alignment can be found in SI Appendix File S3. Bayesian inference analysis was undertaken as described above ($2x10_8$ generations in six Markov chains, sampling every 100_{th} tree and model parameters, 25% burnin), except that we implemented a WAG+G model of sequence evolution selected by ModelGenerator (28).

Selection analyses. To assess the nature of natural selection underpinning the evolution of kallikreins, various site-, branch-, and branch-site maximum likelihood models implemented in CodeML of the PAML (Phylogenetic Analysis by Maximum Likelihood) package (29). To this end, the sequence alignment and consensus tree generated above were used for these analyses. To determine positive selection, the ratio of non-synonymous to synonymous substitutions (ω) was estimated, and nested models M7 (null model) and M8 (alternate model) were compared using a likelihood ratio test (LRT) for determining statistical significance. Bayes Empirical Bayes (BEB) method implemented in the site model 8 (M8) (30) was used to identify amino acid sites under the influence of positive Darwinian selection. Mixed Effect Model of Evolution (MEME) (31) and the Fast Unconstrained Bayesian AppRoximation (FUBAR) analyses implemented in the Datamonkey webserver (32) were performed to determine the effect of episodic and pervasive influence of diversifying selection. For assessing the influence of selection on the eulipotyphlan *KLK1* clade (which incorporates *KLK1* and those arbitrarily annotated as *KLK2* and *KLK3* in *Homo sapiens*), the branch specific two-ratio model (33, 34) was employed by selecting the *KLK1* clade as the foreground branch, and: i) constraining (assuming no positive selection); and ii) relaxing (indicating positive selection) the ω parameter. A LRT test was conducted comparing the null model (neutral evolution) against the alternate model (positive selection). Subsequently, the Adaptive Branch-Site Random Effects Likelihood (aBSREL) approach (35), an improved version of the common branch-site models, was used to identify branches experiencing diversifying selection.

Structural Analyses. Three-dimensional homology models were generated for various tetrapod kallikrein homologs using the Phyre2 server (36), and ConSurf (37, 38) was used for highlighting the evolutionary variability in amino acid sites. PyMOL 2.2 (The PyMOL Molecular Graphics System, Version 2.0, Schrödinger, LLC.) was used for visualization and for the generation of images of the homology models.

Sequence analyses. A sequence alignment of representative eulipoytyphlans was constructed by pruning the dataset described above to only include *KLK1* representatives from *Blarina brevicauda*, *Sorex araneus*, *Condylura cristata*, *Erinaceus europaeus*, *Solenodon paradoxus*, and *Homo sapiens* as an outgroup comparator. The sequences were translated in MEGA v.7 (39), realigned with MUSCLE (25) and then manually inspected. Jalview v.2.10.1 (40)was then used to annotate the alignment with sequence conservation grading. Next, we identified the catalytic triad residues and the five regulatory loops described by Aminetzach et al. (41), and subjected the amino acid residues found within loops 1, 2, 3 and 5 of the various venom-derived and non-venom *KLK1*s to analyses of hydropathy and charge. Regulatory loop 4 was discarded from this analysis due to the short length of the loop (three amino acids long). The hydropathicity and net charge of each loop were calculated for each sequence using the ProtParam tool of the ExPASy

Bioinformatics Resource Portal (https://web.expasy.org/protparam/). Hydropathicity was calculated as the grand average of hydropathicity, and charge was determined based on the presence of the negatively charged amino acids aspartic acid and glutamic acid, and the positively charged amino acids arginine and lysine. Statistical comparisons of the hydropathicity and net charge of venom and non-venom *KLK*s were performed using unpaired two-tailed *t*-tests in Graphpad Prism (La Jolla, USA).

Genomic organisation of kallikreins. For the *de novo* annotation of *KLK* exons and synteny comparisons of mammalian genomic data we used the method described in Koludarov and Aird (42). We extracted exons that corresponded to *KLK* genes according to published annotations, and then used BLAST (BLASTn, e-value of 0.05, default restrictions on word count and gaps) to determine homology of those exons. This step was necessary, since many ab initio annotated *KLK* genes have a varying number of exons. As anticipated, this was an annotation-related artefact and in the final analysis no gene had more than five exons. By removing all unique exons, we created the initial exon database that was used to BLAST-search genomic sequences a wide taxonomic variety of mammalian species used in this study (see Fig. 4). This approach uncovered exons that were absent from published annotations, and by including those newly found sequences in our database, we were able to refine our process and subsequently repeated the BLAST search using the tblastn function of NCBI-BLAST, with e-value cutoffs of 0.01. This process was repeated until no new exons were discovered. We then manually assessed each result and established exon boundaries using Geneious v11 (https://www.geneious.com), relying on previously existing transcriptome-verified exon annotations wherever possible. We paid close attention to variations in exon boundaries between the different *KLK* genes and between different lineages.

Reconstructing the evolutionary origin of venom. Ancestral trait reconstruction of venom in Eulipotyphla was performed with Ape (version 5.2) (43) and Phytools (version 0.4.98) (44) packages in R (45). We treated venom as a trait with a binary distribution (i.e., venomous and non-venomous), and ancestral states were estimated using 'equal rate' (ER) and 'all rates different' (ARD) models, where both forward and backward rates are, respectively, fixed or flexible. The marginal ancestral states (empirical Bayesian posterior probabilities) were estimated for each node in a eulipotyphlan species tree derived from prior studies (46, 47). Finally, a stochastic character mapping analysis (48) was performed for 1,000 simulations, and a trait density map was generated to depict the posterior probabilities of states across all edges and nodes of the species tree.

In vitro assessments of venom function

Serine protease chromogenic assay. We applied a chromogenic assay, using the serine protease-specific chromogenic substrate S-2288 (Cambridge Biosciences), to measure the serine protease activity in the solenodon and snake venoms and that in the solenodon saliva. The reactions were plated in triplicate onto 384-well plates and changes in absorbance were measured at 405 nm for ~30 minutes (kinetic cycle of 21 s) using a FLUOstar Omega microplate reader (BMG Labtech GmbH, Ortenberg, Germany). We initially added 15 µl of diluted venom or saliva (1 µl of venom (1 µg) +14 µl PBS) to the plate, followed by a 3 min incubation at 37 ºC. We then added 15 µl Tris buffer (100 mM Tris, 100 mM NaCl, pH 8.5) and incubated for another 3 min at 37 ºC. Lastly, we added 15 µl of the 6 mM S-2288 chromogenic substrate to each well and set the reaction to run at 37 ºC in the plate reader. A negative control consisting of no venom (15 µl PBS + 15 µl Tris buffer + 15 µl substrate) was used in every experiment, and a positive control, containing 1 µg *Bitis arietans* snake venom, was used to validate the assay. The mean absorbance was plotted against time to compare venom activity with the baseline (negative controls) and positive control readings (SI Appendix Fig. S1). We then subtracted the mean of the negative control readings from each of the venom/saliva and positive control readings and calculated the rate of substrate consumption for each sample by measuring the slope after a specific time interval (t_1~5 min), as follows:

$$Rate_{(Abs/min)} = (A_{405}\ (t_1) - A_{405}\ (t_0))\ /\ time_{(min)}$$

where the absorbances at 405 nm represent the adjusted absorbances from which the negative control has been subtracted. We then plotted the means and the standard error of the mean (SEM) for each sample (n = 3 independent repeats), and performed statistical comparisons of solenodon venom and saliva using an unpaired two-tailed *t*-test in Graphpad Prism (La Jolla, USA).

Phospholipase fluorescent assay. We assessed the continuous phospholipase A_2 (PLA_2) activity of solenodon venom using a previously employed (49) fluorescence substrate assay (EnzChek® Phospholipase A_2 Assay Kit, Cat#E10217, Thermo Scientific, Rochester, NY, USA), measured on a Fluoroskan Ascent® Microplate Fluorometer (Cat#1506450, Thermo Scientific, Vantaa, Finland). A working stock solution of freeze dried *S. paradoxus* venom was reconstituted in a buffer containing 50% MilliQ / 50% glycerol (>99.9%, Sigma) at a 1:1 ratio to and stored at -20°C. Venom (50 ng/µl) was brought up in 12.5 µl 1X PLA_2 reaction buffer (250 mM Tris-HCL, 500 mM NaCl, 5 mM $CaCl_2$, pH 8.9) and plated out in triplicate

on a 384-well plate (black, Lot#1171125, nunc™ Thermo Scientific, Rochester, NY, USA). Triplicates were measured by dispensing 12.5µl quenched 1mM EnzChek® Phospholipase A_2 substrate per well (total volume 25 µl/well) kinetically over 100 mins or until activity had ceased (at an excitation of 485nm, emission 538nm). Purified PLA_2 from bee venom (1 U/ml) was used as a positive control and data was collected using Ascent® Software v2.6 (Thermo Scientific, Vantaa, Finland). Results were analysed (normalised) and graphed in GraphPad Prism 7.0 (GraphPad Prism Inc., La Jolla, CA, USA) displaying mean readings and standard deviations.

Kininogen and fibrinogen degradation gel electrophoresis. We used a degradation SDS-PAGE gel electrophoresis approach (50) to determine whether fibrinogen (Sigma-Aldrich) or high molecular weight kininogen (HMWK) (HK1300, Enzyme Research Laboratories) was cleaved by solenodon venom or saliva. We included the following experimental samples containing: 5 µg of fibrinogen; 5 µg of venom; 5 µg of fibrinogen and 5 µg of venom; 5 µg of saliva; and 5 µg of fibrinogen and 5 µg of saliva. Similarly, we used 5 µg of HMWK instead of fibrinogen and the same setup as above to test whether the degradation of HMWK occurred. Samples were either incubated for 60 minutes at 37 °C before the addition of a reducing protein loading buffer at a ratio of 1:1, or directly mixed with the loading buffer and loaded onto gels (no pre-incubation). The samples were loaded onto 12-well 4-20% Novex Tris-Glycine gels (ThermoFisher) alongside a protein marker (Broad Range Molecular Marker, Promega) and run at 120 V for 1 h. The resulting gels were stained with Coomassie brilliant blue for 1 h, and then destained (4.5:1:4.5 methanol:acetic acid:H2O) for visualisation.

Plasminogen activation assay. We used a kinetic assay, modified from that recently described (51), to indirectly monitor the plasminogen cleavage activity of the solenodon or snake venoms and that in the solenodon saliva. The resulting plasmin activity was then detected via the cleavage of the H-D-Val-Leu-Lys-AMC fluorescent substrate (I-1390, Bachem). The samples were prepared in a final volume of 10 µl in assay buffer (100 mM Tris-HCl pH 7.5, 0.1% BSA) and contained 1 µg of venom or saliva. As a negative control, 10 µl samples containing assay buffer were prepared. In addition, as a positive control, samples containing 600 ng of recombinant Kallikrein (ab117200, Abcam) were included. The samples were pipetted in triplicate onto 384-well plates, followed by the addition of 50 µl/well of a mix containing the plasminogen (SRP6518, Sigma-Aldrich) and fluorescent substrate in assay buffer (final mix concentrations: 200 ng/ml plasminogen and 5 µM substrate). The increase in fluorescence was monitored on a FLUOstar Omega microplate reader for 45 min (kinetic cycle of 9 seconds, 300 cycles) using an excitation wavelength of 355 nm and an emission wavelength of 460 nm. The areas under the curve (AUCs) were calculated for the 0-30-minute interval, which was chosen as the point where the fluorescence in the solenodon venom- and saliva-containing samples had both reached a plateau. Using the AUCs, mean values and SEM were calculated for each sample (n = 3 independent replicates), and then statistical comparisons of solenodon venom and saliva were undertaken using an unpaired two-tailed *t*-test in Graphpad Prism (La Jolla, USA).

Identification of plasminogen activating toxins via nanofractionation. Solenodon venom was fractionated by liquid chromatography (LC) in parallel with at-line nanofractionation, with subsequent mass spectrometric analyses for the identification of bioactive components. For LC separation, we used a Shimadzu UPLC system ('s Hertogenbosch, The Netherlands) and a Shimadzu SIL-30AC autosampler for the injection of 50 µl of 5 mg/mL venom. A total flow rate of 500 µl/min was provided by two Shimadzu LC-30AD pumps. For the separation of venom we used a 250x4.6 mm Waters Xbridge Peptide BEH300 C18 analytical column with a 3.5-µm particle size and a 300-Å pore size, and the Shimadzu CTD-30A column oven was kept at 30°C. The mobile phase A comprised of 98% H_2O, 2% acetonitrile (ACN) and 0.1% formic acid (HFo), and mobile phase B comprised of 98% ACN, 2% H_2O and 0.1% HFo. The following gradient was used: an increase of mobile phase B from 0% to 50% in 20 min, followed by an increase from 50% to 90% B in 4 min and a 5 min isocratic separation at 90% B. Subsequently, the starting conditions were reached at 1 min, followed by column equilibration for 10 min at 0% B. There was a post-column flow split in a 1:9 ratio. 90% of the fraction was sent to a nanofraction collector, a modified Gilson 235P autosampler. The other 10% was sent to a Shimadzu SPD-M30A photodiode array detector and a maXis impact quadrupole–time-of-flight (qTOF) mass spectrometer (Bruker, Bremen, Germany). The mass spectrometer was equipped with an electrospray ionization source (ESI) and was operated in positive-ion mode. The following ESI source parameters were used: source temperature 180°C, capillary voltage 4.5 kV, nebulizer at 0.4 Bar and dry gas flow 4 l/min. Full MS spectra were recorded in a m/z 50–3000 range at 1 spectrum/s rate. Bruker Compass software was used for controlling the instrument and data analysis. The nanofractions were collected in a 384-well plate in a column serpentine-like fashion using Ariadne, in-house customs software, which allowed for 6-s nanofractions to be collected into black 384-well plates (Greiner Bio One, Alphen aan den Rijn, The Netherlands) with a maximum of six plates in one sequence. 350 wells of the 384-well plate were collected for each chromatographic run. The plates were evaporated overnight after nanofractionation, for approximately 16 h using a Christ Rotational Vacuum Concentrator (Salm en Kipp, Breukelen, The

Netherlands) RVC 2-33 CD plus. After freeze-drying, the plates were stored at –20°C until further use for either tryptic digestion or for bioassaying.

The previously developed plasmin assay referred to above (Zietek et al. 2018) was adapted in order to screen for plasminogen activating activity on the resulting nanofractionated plates. The assay was performed by making a bioassay mix containing 200 ng/ml plasminogen, 5 µM of the fluorogenic substrate H-D-Val-Leu-Lys-AMC (I-1390, Bachem) dissolved in 100 mM TRIS-HCl buffer (pH 7.5), containing 0.1% BSA (w/v). The mixture was prepared by adding the same volumes of enzyme and substrate solutions in to the buffer. Immediately after preparation, the mixture was dispensed on to the nanofractionated plates using a Multidrop 384 reagent dispenser (Thermo Scientific, Ermelo, The Netherlands). A VarioSkan LUX microplate multimode reader (Thermo Scientific, Ermelo, The Netherlands) was then used to measure the fluorescence of each well kinetically at 380 and 460 nm excitation and emission wavelengths, while the bandwidth was 12 nm. The temperature inside the platereader was kept at 37 °C throughout. The measurements consisted of 30 cycles, resulting in the construction of a kinetic curve and its corresponding slope, which was plotted against the time of the collected fractions, resulting in a bioactivity chromatogram.
We identified proteins in the wells responsible for conferring plasminogen activating activity via nanoLC-MS/MS analysis of tryptic digests. We used an UltiMate 3000 RSLCnano system (Thermo Fisher Scientific, Ermelo, The Netherlands) for NanoLC separation of the tryptic digests, with full-loop injection mode run by the autosampler. A 1 µl injection volume was used by the autosampler, and after injection the samples were separated on an analytical Aqua C_{18} capillary column (150 mm x 75 µm) packed in-house (3 µm particle size and 200 Å pore diameter; Phenomenex, Utrecht, The Netherlands). The mobile phases consisted of eluent A (98% H_2O, 2% ACN, 0.1% HFo) and eluent B (98% ACN, 2% H_2O, 0.1% HFo). The following gradient was used for separation: 2 min isocratic at 5% solvent B, linear increase to 80% solvent B in 15 min, 3 min isocratic at 80% solvent B, down to 5% solvent B in 0.5 min and finally column equilibration for 9 min. The column oven was kept at 30°C throughout. A Variable Wavelength Detector set at 254 nm followed by a Bruker Maxis q−TOF mass spectrometer (Bruker, Bremen, Germany) were used for detection. The mass spectrometer had an electrospray ionization (ESI) source and was operated in positive-ion mode. The parameters for the ESI source of the MS instrument comprised of: capillary voltage 4.5 kV, gas flow 10 l/min and source temperature 200 °C. Mass spectra were obtained in the range of 50 to 3000 m/z and at 1 spectrum/s. Data-dependent mode was used to obtain MS/MS spectra by using 35-eV collision energy in the CID collision cell. Bruker Compass software was used for the instrument control and data analysis. Finally, we used MASCOT (Matrix Science, London, United Kingdom) for protein identification of the analysed tryptic digests, via a search against translations of the genes annotated in the *Solenodon paradoxus* genome. The following search parameters were used; instrument type: ESI-QUAD-TOF, digestion enzyme: semiTrypsin allowing for one missed cleavage, fixed modification: carbamidomethyl on cysteine, variable modifications: amidation (Protein C-term) and oxidation on methionine, mass tolerance: ± 0.05 Da fragment and peptide mass tolerance: ± 0.2 Da.

Details of all peptide/protein spectrum matching for the identification of bioactive proteins are displayed in SI Appendix File S4. The raw data have also been deposited to ProteomeXchange (http://proteomecentral.proteomexchange.org) with the ID PXD009593 via the Mass Spectrometry Interactive Virtual Environment (MassIVE, https://massive.ucsd.edu/) with the accession number MSV000082307.

Electrophysiology. We performed patch-clamp electrophysiology experiments using TE671 human rhabdomyosarcoma cells endogenously expressing embryonic muscle-type nicotinic acetylcholine receptors (nAChR) (52) and $Na_v1.7$ voltage gated sodium channels (VGSC) (53, 54). Cells were cultured in 4.5 g/l glucose Dulbecco's modified Eagle's Medium (DMEM, Sigma) supplemented with 10% foetal bovine serum, 2 mM glutamine, 10 IU/ml penicillin and 20 µg/ml streptomycin (Sigma). Cells were plated in 35 mm Petri dishes over heat-sterilised sections of glass coverslips in 2 ml of DMEM and kept in a 36.5°C incubator with 5% CO_2. Locust (*Schistocerca gregaria*) primary neurons (natively expressing insect neuronal nAChR) were dissected from the mushroom bodies of 6_{th} instar locusts and dissociated by incubating in Rinaldini's saline (135 mM NaCl, 25 mM KCl, 0.4 mM $NaHCO_3$, 5 mM D-glucose, 5 mM HEPES, pH 7.2 with NaOH, with 2 mg/ml collagenase and 0.5 mg/ml dispase) for 15 minutes at 36.5°C. Cells were gently triturated with a 200 µl Gilson pipette tip and plated over poly-L-lysine coated glass coverslips in 5:4 DMEM:Schneider's medium and incubated for 12 hours at 36.5°C with 5% CO_2. Patch-clamp experiments were performed within 36 hrs of dissection.

Patch pipettes with a resistance 5-7 MΩ were pulled from thick-walled borosilicate glass (World Precision Instruments, USA) using a P-97 Flaming/Brown micropipette puller (Sutter Instrument Co., USA) and filled with a caesium pipette solution (140 mM CsCl, 10 mM NaCl 1 mM $MgCl_2$, 11 mM EGTA and 5 mM HEPES, pH 7.2 with CsOH). The bath solution for TE671 cells was 135 mM NaCl, 5.4 mM KCl, 1 mM $CaCl_2$, 1 mM $MgCl_2$, 5 mM HEPES and 10 mM D-glucose (pH 7.4 with NaOH), and for locust neurons was 180 mM NaCl, 10 mM KCl, 2 mM $CaCl_2$, 10 mM HEPES, pH 7.2. Whole-cell currents were monitored using an Axopatch 200A (Axon instruments, USA) patch-clamp amplifier and recorded using WinWCP V4.5.7 software (Dr John Dempster, University of Strathclyde). Venom and agonist were applied to cells using a DAD-12 Superfusion

system (Adams and List Associates, USA) fitted with a 100 µm polyamide coated quartz output tube with a solution exchange time of 30-100 ms. Control responses and those in the presence of venom were obtained on the same cell and repeated for n≥4 separate cells. Series resistance was compensated by 75% to minimize any voltage errors, and data were filtered at 10 kHz. Data were analysed using GraphPad Prism 7.

In vivo assessments of venom function

Locust toxicity assay. Fifth instar desert locusts (*Schistocerca gregaria*) were injected at 0.05 ml/g with venom at various doses (0.1, 1, 10 and 50 µg/g solenodon venom, n=4 per dose) in sterile filtered (0.22 µm Starstedt) insect Ringer's saline (4.35 mM $CaCl_2$(+2H_2O), 3.81 mM $NaHCO_3$, 4.3 mM KCl and 0.17 mM NaCl, adjusted to pH 7.2 with NaOH). The injection site was between the 1st and 2nd ventral tergite, towards the thorax, and was performed using a 300 µl Micro-Fine insulin syringe. Ringer alone was used for controls (n = 4). Injected locusts were placed individually in ventilated livefood tubs and kept at 26 °C. Their status was recorded at 24, 48 and 72 hours post injection as either alive, incapacitated (unable to self-right within 10 s when placed on back) or dead.

Centipede toxicity assay. To test the toxicity of solenodon venom against centipedes we injected juvenile giant centipedes (*Ethmostigmus rubripes*; 4-5 cm total length) bred in the lab from specimens collected in Brisbane, Queensland, Australia, with 2 µl of venom at various doses (20 µg/g and 100 µg/g solenodon venom in insect Ringer's saline, n = 5). Negative controls were injected with Ringer's saline only (n = 5). The injection was done using a 1 ml 29G insulin syringe into the 5th trunk segment. Injected centipedes were placed individually in ventilated food tubs with a piece of wet paper for moisture, but no substrate, and kept at 25 °C. Their status was recorded at 30 mins, 60 mins and 24 hours post injection as either alive, incapacitated (unable to respond to contact from forceps) or dead.

Pulse-oximeter measurements of mice. To measure the physiological responses of mice dosed with solenodon venom in a non-invasive manner, we used a MouseOx pulse-oximeter monitoring system (MouseOx, Harvard Apparatus), coupled to MouseOxPlus software (Starr Life Sciences) for data collection and analysis. The experiment consisted of groups of male 20 g CD1 mice (Charles River) receiving either 500 µg solenodon venom (n=3) or PBS, pH 7.2 (n=3) via intravenous injection (100 µl dose/animal via the tail vein). Throughout the experiment, we collected the following physiological parameters: oxygen saturation (% oxygen), pulse rate (beats per minute), respiration rate (breaths per minute) and pulse distension (mmHg). The data was collected via a monitoring collar attached to the recording device, which was applied to the neck of each mouse and held in place until eight values were measured on the resulting trace (typically over a period of ~1 min). To ensure data robustness, readings were only retained where 'error' and 'activity' were zero. Each experimental animal was subjected to five independent measurements (each consisting of the eight values mentioned above) at different timepoints: at baseline (prior to the administration of venom or saline) then at 1 min, 15 min, 30 min and 45 min post-administration. For each time point, mean readings for each experimental animal were calculated, prior to the calculation and plotting of group means and standard deviations expressed as the percentage of baseline readings. This *in vivo* animal experiment was conducted using protocols approved by the Animal Welfare and Ethical Review Boards of the Liverpool School of Tropical Medicine and the University of Liverpool, and performed in specific pathogen-free conditions under licenced approval of the UK Home Office, in accordance with the Animal [Scientific Procedures] Act 1986 (UK) and institutional guidance on animal care.

Mean arterial blood pressure in rats. The effect of *S. paradoxus* venom on blood pressure was examined in anaesthetized rats, as described previously (55). We first anaesthetized male rats (Sprague-Dawley; 250-320 g) with 100 mg/kg ketamine and 10 mg/kg xylazine (i.p.). A midline incision was made and cannulae inserted into the trachea, jugular vein and carotid artery to enable artificial respiration if required, administration of venom, and recording of arterial blood pressure, respectively. The carotid artery cannula was connected to a PowerLab/400 system via a Gould Statham P23 pressure transducer. Before the injection of venom, blood pressure was allowed to stabilize for at least 10 min. Body temperature was maintained at approximately 37°C using an overhead lamp and heated table. Venom (1 mg/kg; n=5) was administered through the jugular vein and flushed with saline (0.2 ml). Control traces were obtained by flushing only with saline. Animals were assigned randomly to experimental groups, and the experimenters were not blinded to the condition. This procedure was approved by the Monash Animal Research platform (MARP) Animal Ethics Committee, Monash University, Australia (#MARP/2017/147).

Dietary analyses of wild solenodons

To assess the contribution of vertebrate prey to the diet of *S. paradoxus*, fieldwork was carried out in the Dominican Republic during two seasons: in January to February 2015 (dry season) and July to August 2015 (wet season). Fieldwork was undertaken with permission from the Secretaría de Estado de Medio Ambiente y Recursos Naturales, Dominican Republic. Samples were collected in the Bahoruco-Jaragua-Enriquillo

UNESCO Biosphere Reserve region in Pedernales Province along the southwestern edge of Parque Nacional Sierra de Bahoruco at 300-400 masl (~18°08' N, 71°39' W). The natural vegetation of the sampled area is mid-elevation broadleaf forest, although increasing anthropogenic impacts on this landscape have created a mosaic agriculture-forest habitat consisting of unmanaged pastures, small-scale mixed cropland, and primary and secondary forest fragments. Abundant plant families include Malvaceae, Euphorbeaceae, with the leguminous species *Acadia macracantha* and *Prosopis juliflora* common in disturbed areas. Geologically, Miocene limestone karst dominates the landscape, often serving as burrows for solenodon family groups and supplying potential food items such as land snails and arachnids. We identified active solenodon foraging sites using “nose-pokes”, which are diagnostic conical holes in the soil and leaf litter made by the solenodon’s probing long nose. These tracks are often accompanied by diagnostic tail impressions and diggings made by the animal’s robust forelimbs. Solenodon faeces can be readily identified by the presence of abundant chitinous millipede exoskeletons in the faecal matrix, and the invertebrate prey diversity of *S. paradoxus* has previously been described (56). To detect vertebrate prey, we collected fresh faeces (< 2 days) opportunistically when encountered near foraging sites and active burrows. Individual collected samples were flash preserved in 95% ethanol for <24 hours initially, then decanted with the ethanol completely removed by drying with silica beads. The faeces were then stored dry until later analysis.

We collected a total of 64 solenodon faecal samples, with 40 collected in the dry season and 24 in the wet season. DNA was extracted using a PowerFecal® DNA Isolation Kit (MO BIO Laboratories Inc, Carlsbad, CA; Catalog No. 12830-50) to facilitate removal of PCR inhibitors specific to faeces. We then used previously designed vertebrate primer sets for 12S (57) and 16S (“16smam”) (58) ribosomal genes to probe for the presence of vertebrate prey DNA in solenodon faeces. Resulting DNA was sequenced on an Ilumina MiSeq machine at the National High-throughput DNA Sequencing Centre of Denmark. DNA operational taxonomic unit (OTU) sequences were then compared to Genbank’s non-redundant nucleotide and the Barcode of Life Data Systems (BOLD) databases to identify prey items above an 85% identity threshold. To calculate the frequency of occurrence of vertebrate prey, we summed the presence of a food item across all 64 samples (e.g. following the approach of (59)) and calculated the percent frequency occurrence using the following formula:

$$\%FC_i = \left(\frac{N_i}{N}\right) x\ 100\%$$

where N is the total number of faecal samples considered and N_i is the number of samples containing the food item *i* (60).

As the identification of vertebrate prey items was reliant upon available reference sequences in Genbank and BOLD, we were unable to identify OTUs to the genus and species level, and our resulting frequency of occurrence value of 12.3% is therefore a conservative estimate. In addition, the sampled region represents just one of many biomes inhabited by *S. paradoxus* across Hispaniola, and due to the aforementioned impact of anthropogenic disturbance on this region, it is possible that this generalist species may alter its diet – and therefore the proportion of vertebrate prey – to reflect local conditions. As Hispaniola experienced massive vertebrate extinctions in the Mid-Holocene and historic period, it is also possible that their preferred prey items have been lost, forcing solenodons to decrease vertebrate protein in their diet. However, as introduced mammals have largely replaced native mammals in body size (61), it is unclear whether solenodons would have experienced an overall decline in the availability of vertebrate prey.

SI Appendix Figures

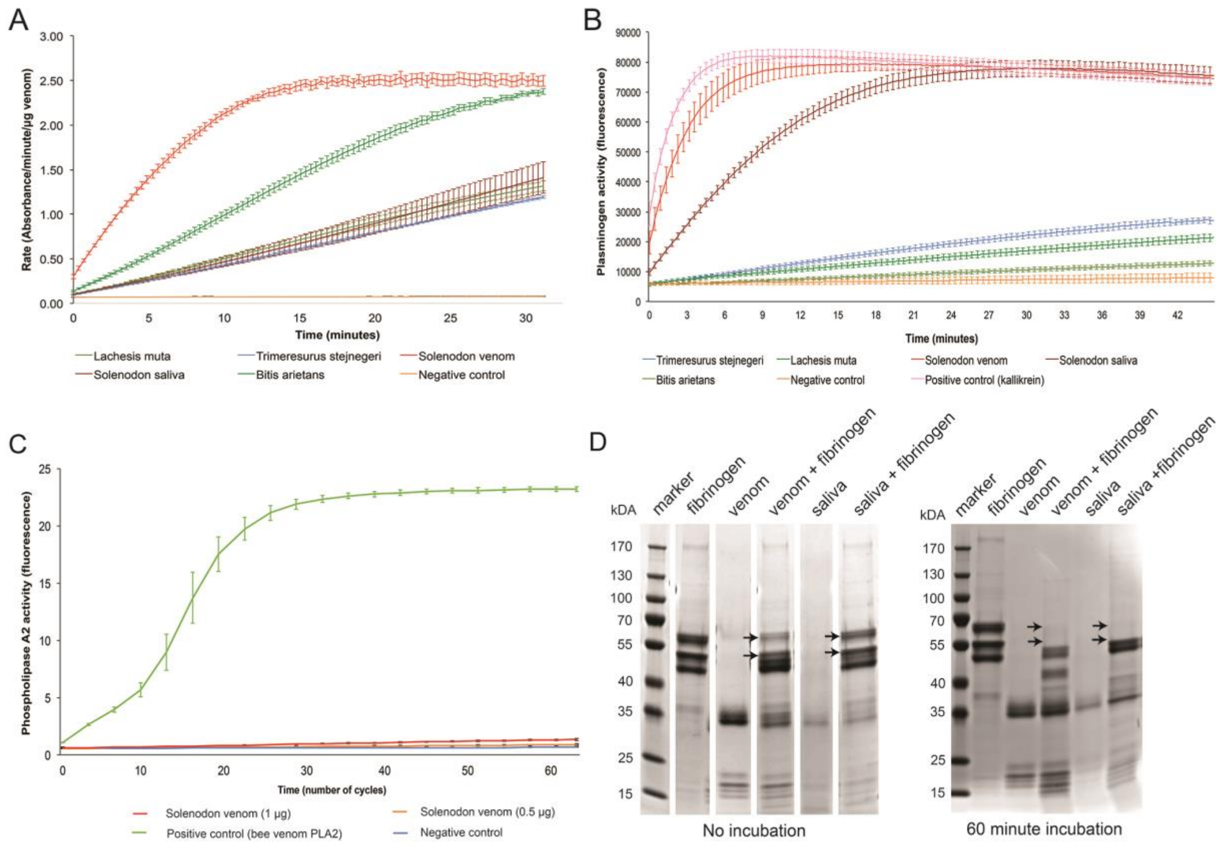

Fig. S1. The *in vitro* activity of *S. paradoxus* venom. Raw plotted data used to calculate areas under the curve displayed in Fig. 2 for the chromogenic serine protease assay (**A**) and fluorescent plasminogen activating assay (**B**). **C)** Solenodon venom shows no evidence of enzymatic phospholipase activity as measured by fluorescent enzyme assay. **D)** Solenodon venom and saliva both degrade the alpha and beta chains of fibrinogen (indicated by arrows), but not in a potent manner, as incubation is required to detect extensive degradation. The data displayed in **A-C** represent every third reading collected (to aid the display of data) and each data point represents the mean of triplicate measurements, with error bars representing SEM.

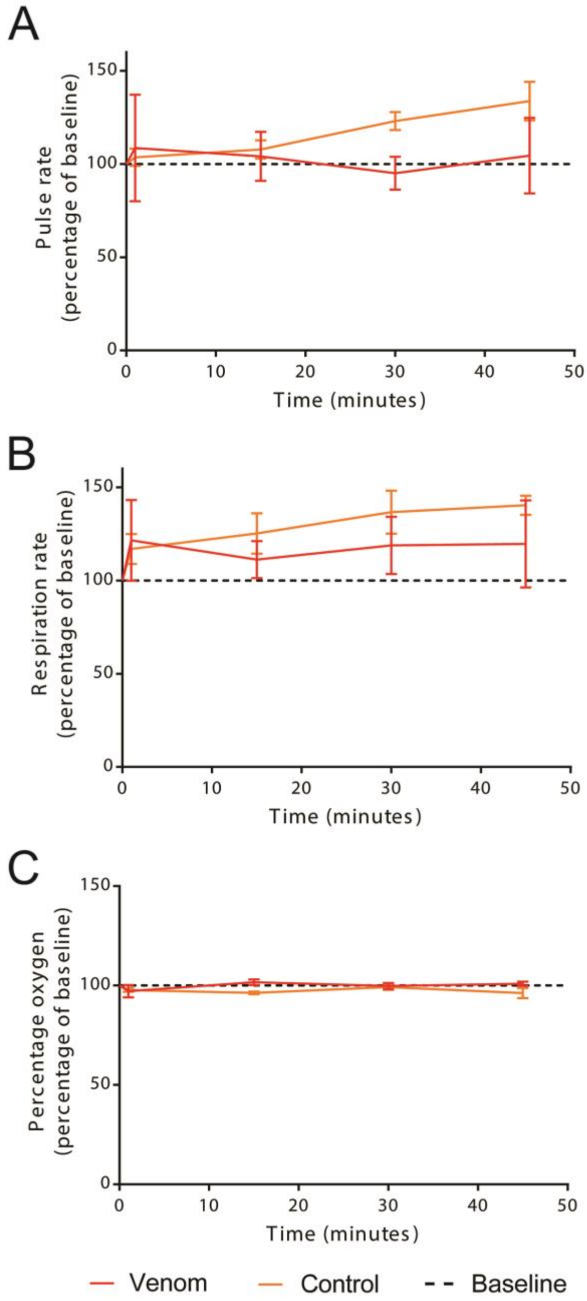

Fig. S2. Pulse oximeter measurements collected from mice intravenously administered with solenodon venom. The three line charts compare the pulse rate (**A**), respiration rate (**B**) and percentage oxygen (**C**) collected from mice that received either solenodon venom (25 mg/kg) or saline control. The data is displayed as percentage changes from baseline readings collected prior to injection (dashed lines). For each group n=3, with data points representing the mean of the three readings, and error bars representing standard deviations.

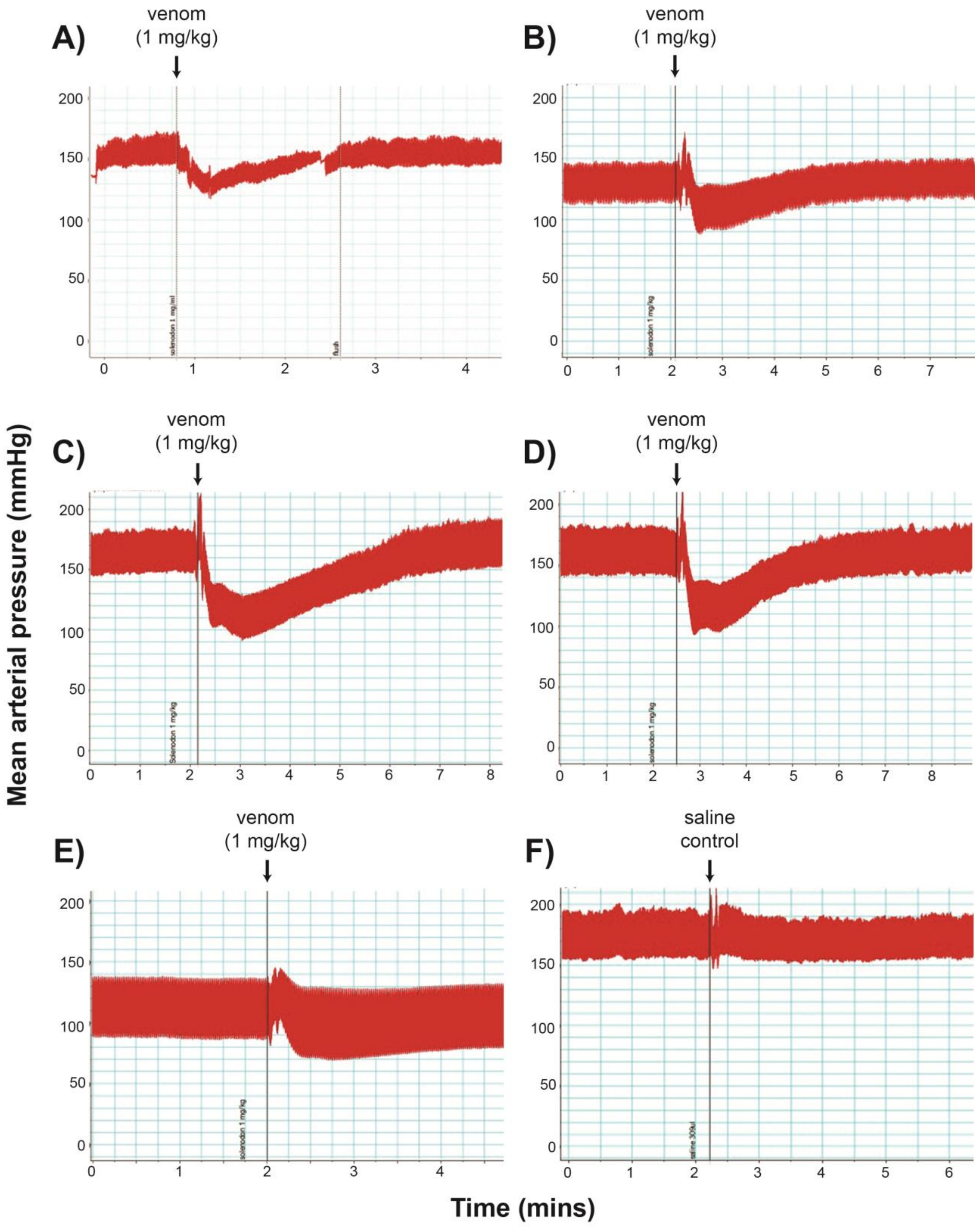

Fig. S3. Solenodon venom causes a transient depressor effect on the mean arterial blood pressure of the anaesthetised rat. **A-E)** The data displayed is traces from the five individual experimental animals that received 1 mg/kg solenodon venom. **F)** Representative trace from one of the experimental control animals treated with saline only (no venom).

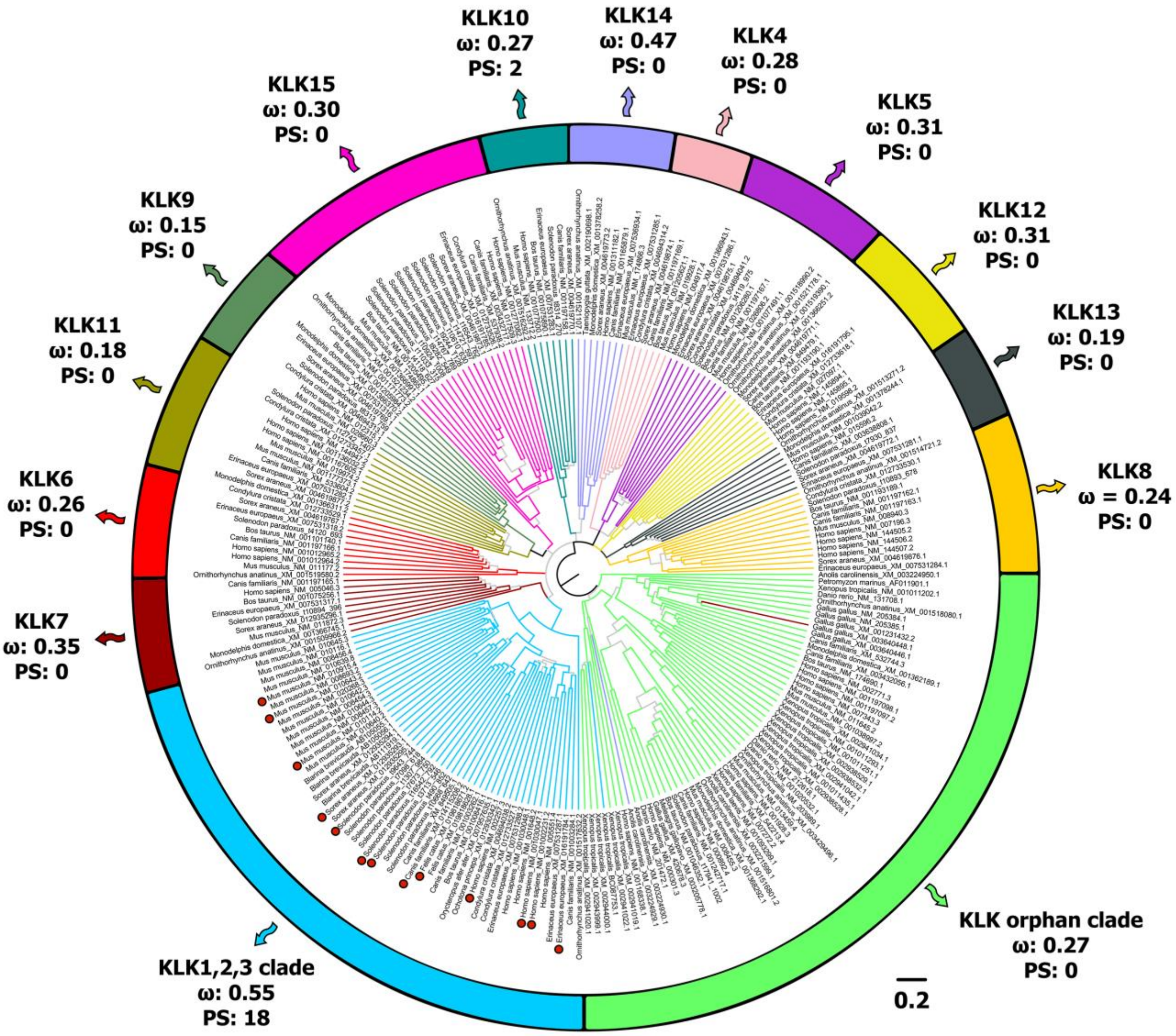

Fig. S4. Bayesian phylogeny of tetrapod kallikreins. A midpoint rooted Bayesian phylogeny of kallikreins is presented, where branches with Bayesian posterior probability (BPP) of less than 0.95 are shaded grey. Estimates of omega (ω) parameter for the respective lineages, computed by the site model 8, are indicated along with the total number of positively selected sites (PS) identified by the Bayes Empirical Bayes (BEB) method. Branches indicated by a red circle were identified by the Adaptive Branch-Site Random Effects Likelihood (aBSREL) analysis as evolving under the influence of positive selection.

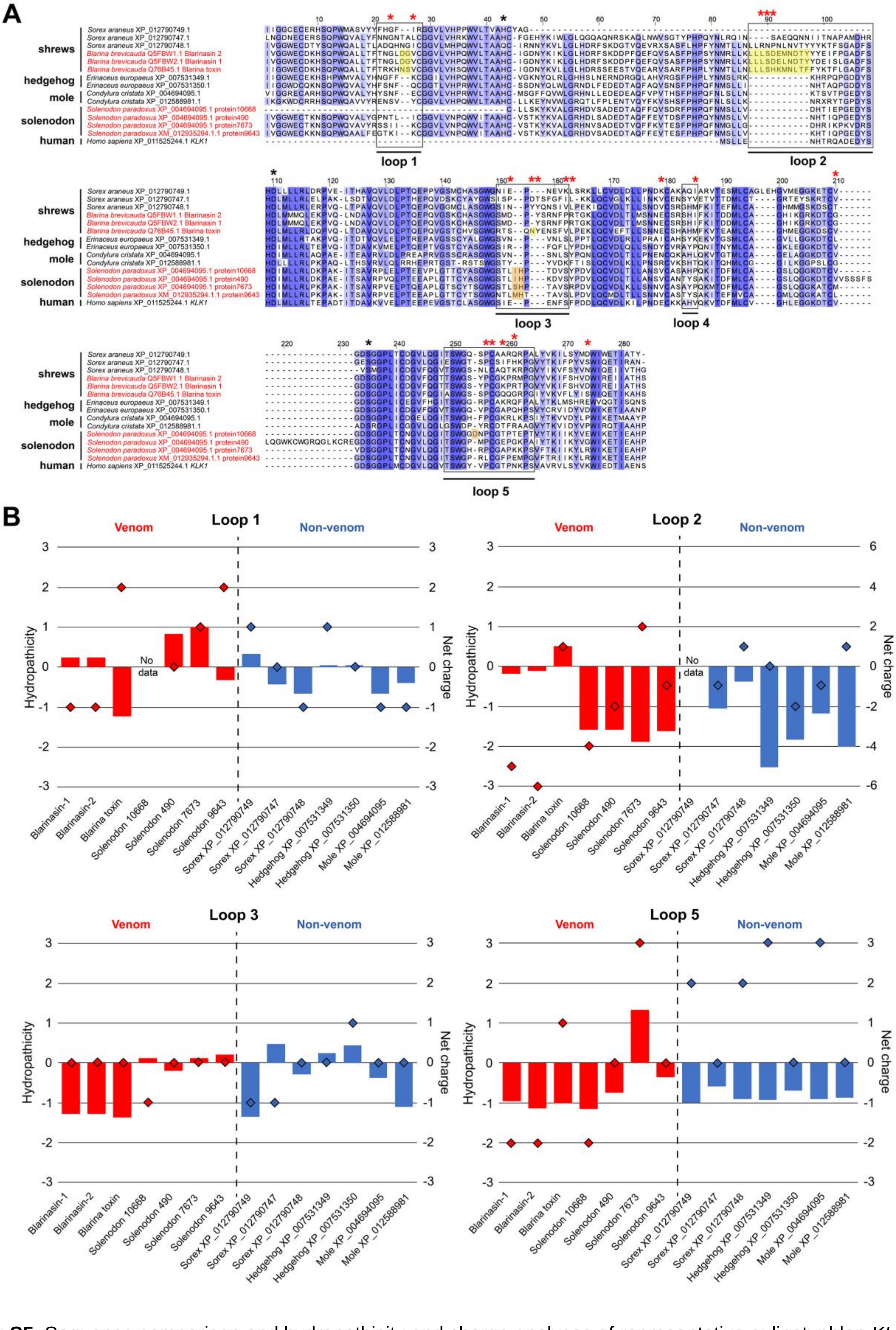

Fig S5. Sequence comparison and hydropathicity and charge analyses of representative eulipotyphlan *KLK1* amino acid sequences **A)** Amino acid sequence alignment of representative eulipotyphlan *KLK1s*. Red labels highlight those sequences identified in venom. Amino acid residues are coloured in a graded manner by sequence conservation. The five regulatory loops previously detailed by Aminetzach et al. (41) are

highlighted by black boxes (note that Aminetzach et al. have inadvertently mislabelled loop 1 as loop 2 and vice versa in their main text of their manuscript), and previously described insertions in *Blarina* venom kallikreins (41, 62, 63) are shaded yellow. Orange shading highlights novel insertions within regulatory loops observed in *Solenodon* venom *KLK1*s. Black asterisks highlight the previously proposed catalytic triad residues (41) and red asterisks highlight positively selected sites detected by the Bayes Empirical Bayes approach (see SI Appendix Table S4). *Homo sapiens KLK1* is included as an outgroup comparator. **B)** Hydropathicity and net charge analyses of the regulatory loops of representative eulipotyphlan *KLK1* amino acid sequences. Bar charts show comparisons of hydropathicity (grand average of hydropathicity), and diamonds show comparisons of net charge. Data from kallikreins identified from venom, either in this study or prior studies (62, 63), are coloured red, and those from non-venomous taxa in blue. Data is not displayed for regulatory loop 4 due to the short length of this loop (three amino acids long). Note the change in scale for net charge in loop 2.

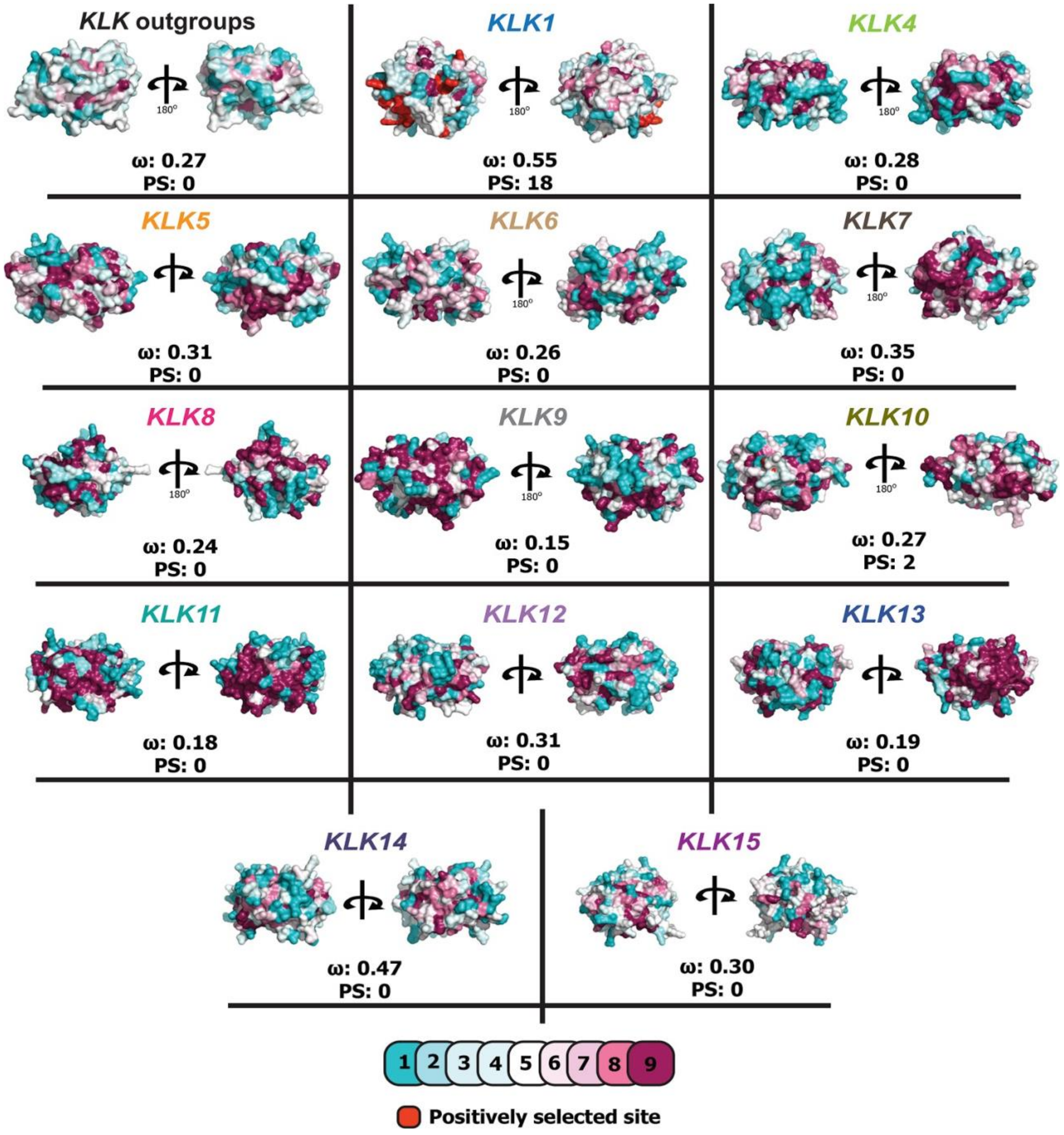

Fig. S6. Three-dimensional models of various tetrapod kallikrein homologs depicting positively selected sites (highlighted in red). The colour code provided highlights evolutionarily conserved and variable amino acid positions, indicated by a gradient of purple to blue.

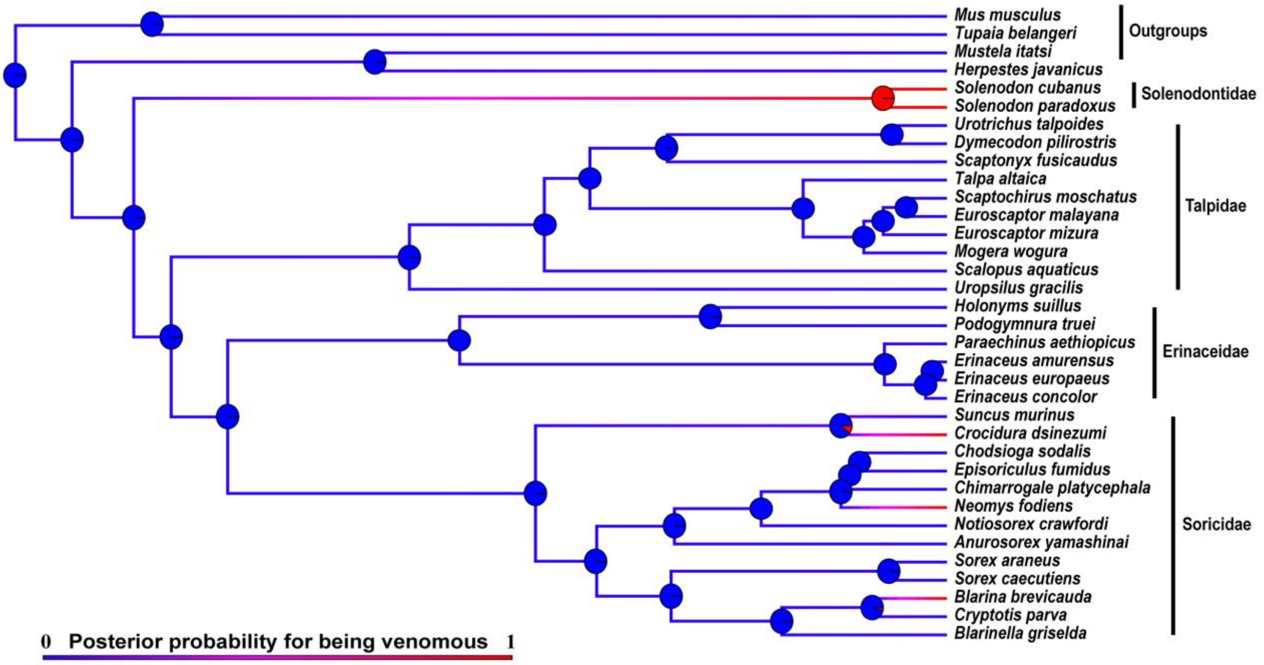

Fig. S7. A eulipotyphlan species tree overlaid with a trait density map for venom generated by stochastic character mapping. The posterior probability (0 to 1) of each branch and node to be venomous is depicted by a colour gradient or pie charts, where blue represents non-venomous and red represents venomous.

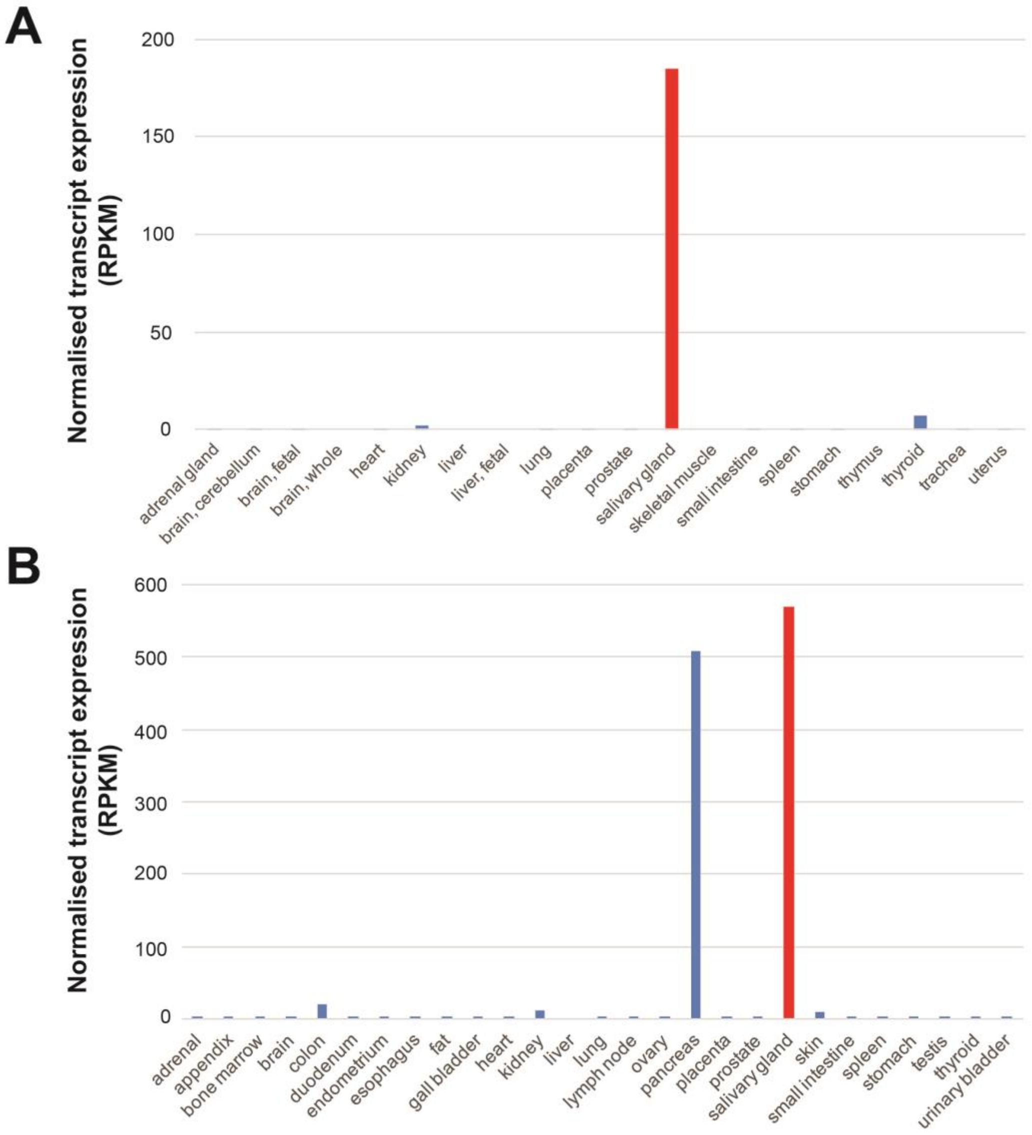

Fig. S8. Comparative relative tissue expression of *KLK1* in *Homo sapiens*. **A)** Comparison of tissue expression based on RNA sequencing of total RNA from 20 human tissues. Data from BioProject PRJNA280600 (64). **B)** Comparison of tissue expression based on RNA sequencing of 27 tissues from 95 human individuals. Data from BioProject PRJEB4337 (65). Data expressed as Reads Per Kilobase of transcript per Million mapped reads (RPKM). Salivary gland samples are highlighted in red.

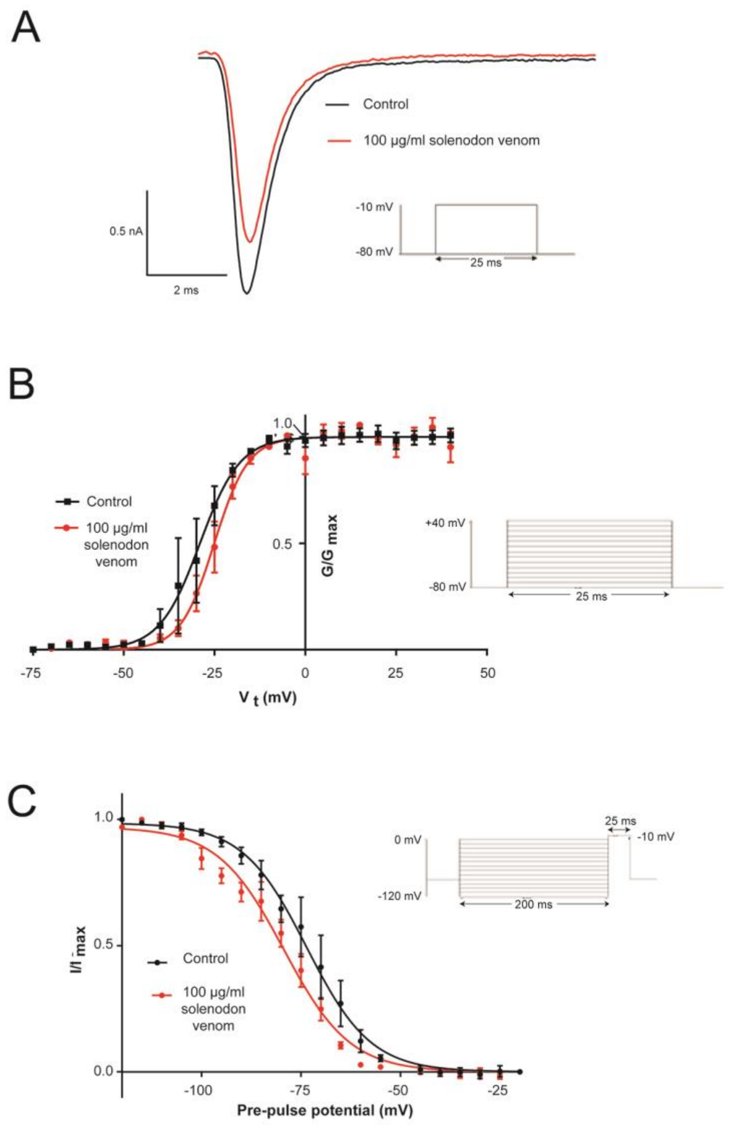

Fig. S9. Assessing the potential activity of solenodon venom on voltage gated sodium channels. **A**) Effects of solenodon venom on voltage gated sodium channels (VGSC) endogenously expressed by TE671 cells. Whole-cell current traces in the absence and presence of 100 µg/ml solenodon venom are displayed (n=6). Inset: voltage protocol for the step depolarisation to -10 mV from a holding potential of -80 mV. **B)** Conductance-voltage relationship in the absence and presence of 100 µg/ml solenodon venom. Current data were transformed into and normalised to maximum peak conductance (±SEM) and the curves displayed are fits of a Boltzmann's equation that enabled estimation of half activation voltages ($V_{50.act}$) (see also SI Appendix Table S8). Inset: Step depolarisations from –80 to +40 mV in 5 mV increments for 25 ms from -80 mV holding voltage. **C)** Voltage dependence of steady-state inactivation of VGSC expressed by TE671 cells in the absence (control) and presence of 100 µg/ml solenodon venom. Normalised peak currents plotted as a function of the pre-pulse potentials and fitted with a Boltzmann sigmoidal relationship for the computation of the $V_{50.inact}$ value (n=4) (see also SI Appendix Table S8). Inset: The voltage protocol showing pre-pulse potentials from -120 mV to -20 mV for 200 ms before a -10 mV depolarisation step for 25 ms.

SI Appendix Tables

Table S1. Comparison of genome assembly statistics between the top assembly from Grigorev et al. (1) (assembly A) and the resulting assembly from this work.

Assembly metric	Grigorev et al. 2018	This paper
Contig N50 (bp)	54,944	236,671
Scaffold N50 (bp)	555,585	407,682
Percent Ns	0.06	0.02
Partial BUSCOs (%)	17%	4.7%
Complete BUSCOs (%)	74%	92.9%

Table S2. Summary of protein identifications from the proteomic experiments undertaken on *S. paradoxus* venom and saliva by shotgun (trypsin digested), decomplexed (trypsin digested prefractionated venom) and top-down approaches. Support and approximate comparisons of abundances between venom and saliva for each identified protein is given as the percent of all confidently (>95 %) assigned tryptic peptides assigned to each by Protein Pilot (shotgun), the number of validated tryptic peptides identified by X!Tandem (decomplexed), or the intensity of the intact precursor protein peak (top-down). Detailed information on Peptide/Protein Spectrum Matching (PSM/PrSM) can be found in SI Appendix File S1 and in the ProteomeXchange dataset (deposition accession PXD009593).

Protein annotation	Genome match	Venom			Saliva	
		Shotgun (percent 95% confidence peptides)	***Decomplexed*** (number of validated peptides)	***Top-Down*** (Intensity from XIC)	***Shotgun*** (percent 95% confidence peptides)	***Top-Down*** (Intensity from XIC)
Kallikrein-1	9643	21.08	11		10.80	
Kallikrein-1	7673	16.27	8	1.73E+07	5.16	8.09E+04
Kallikrein-1	10668	11.75	5	1.03E+07	4.23	2.40E+06
Kallikrein-1	490	20.78			8.92	
Kallikrein-1	1307	11.45			7.51	
Kallikrein-1	7098	6.33			4.69	
Kallikrein-1	16542	6.93				
Zymogen granule protein	15655	1.51			1.41	
Deoxyribonuclease	13375	1.20			0.47	
Neutrophil gelatinase associated lipocalin	13014	0.60			0.94	
Olfactory receptor 52Z1-like protein	5016	0.30			0.94	
Lysosomal alpha mannosidase	3805	0.30				
Beta hexosaminidase	4900	0.30				
Complement factor H	15966	0.30				
PC esterase domain containing protein	17726	0.30				
PC esterase domain containing protein	14842	0.30				
PC esterase domain containing protein	1814	0.30				
Serum albumin	1900				2.82	
Protein S100-A7	10247				0.94	
Protein S100-A8	9574				2.35	
Protein S100-A9	9358				1.88	
Protein S100-A12	9355				1.41	
Adenosine deaminase	5185				2.35	
Polymeric Ig receptor	640				1.88	

BPI fold containing family B member	9017	1.88
BPI fold containing family B member	11740	0.47
Lactotransferrin	18056	1.41
Alpha-2-macroglobulin	1093	0.94
Histone H2A	14235	0.47
Histone H2A	15986	0.47
Histone H2A	10906	0.47
Histone H2A	15997	0.47
Histone H2A	3965	0.47
Histone H2A	9054	0.47
Histone H2A	17930	0.47
Histone H2A	12020	0.47
Histone H2B	10256	1.41
Histone H2B	15999	1.41
Histone H2B	5402	1.41
Histone H2B	10283	1.41
Histone H2B	14410	1.41
Histone H2B	8727	1.41
Histone H2B	831	1.41
Histone H2B	14957	1.41
Histone H2B	3960	1.41
Histone H2B	10284	0.94
Histone H3	14411	0.94
Histone H3	10280	0.94
Histone H3	10266	0.94
Histone H4	10254	0.94
Histone H4	7623	0.94
Histone H4	17563	0.94
Mucin	3632	0.94
Mucin	5939	0.94
Uromodulin	16702	0.94
Cystatin A	2910	0.94
Zinc alpha-2-microglobulin	7038	0.94
Serotransferrin-like	4139	1.41
Lysozyme C	6917	0.47
Immunoglobulin J chain	12360	0.47
Annexin A1	8846	0.47
Beta-actin-like protein	15699	0.47
Actin cytoplasmic	5124	0.47
Actin cytoplasmic	15125	0.47

Cornifin alpha	770	0.47
Cornifin beta	771	0.47
Submaxillary mucin like protein	12133	0.47
Uncharacterised Ig protein	18037	0.47
Ig kappa chain	6933	0.47
Ig kappa chain	6930	0.47
Ig kappa chain	17843	0.47
Immunoglobulin superfamily member 3	4688	0.47
Immunoglobulin light chain	4687	0.47
Immunoglobulin light chain	15854	0.47
Cathepsin G	18080	0.47

Table S3. Identification of bioactives in solenodon venom that exhibit plasminogen activating activity. Selected wells of the plasminogen activating assay that exhibited activity (see Fig. 3C) were subjected to tryptic digestion and mass spectrometric analyses. Matches to the translated *S. paradoxus* genome database are displayed where coverage was ≥5% and protein score ≥50. Note that proteins exhibiting similarity with serum albumin were detected in each well, as the result of bovine serum albumin being present in the bioassay buffer (see Methods). In total, three distinct *KLK1* venom proteins were identified (Genome IDs 490, 10668, 9643) in six different wells. Detailed information of Peptide Spectrum Matching can be found in SI Appendix File S4.

Well	Retention time	Genome match	Genome annotation	Coverage (%)	Protein score
**H11**	18.5	KLK1|XP_004694095.1 **|protein-490**	***KLK1***	26	494
		KLK1|XP_004694095.1 **|protein-10668**	***KLK1***	42	413
		ALB|XP_004681276.1 |protein-1900	Serum albumin	8	356
**P11**	19.4	ALB|XP_004681276.1 |protein-1900	Serum albumin	6	184
		LOC101548172|XM_012935294.1 **|protein-9643**	***KLK1***	5	74
**I12**	20.2	ALB|XP_004681276.1 |protein-1900	Serum albumin	9	186
		LOC101548172|XM_012935294.1 **|protein-9643**	***KLK1***	5	77
**A13**	21.2	ALB|XP_004681276.1 |protein-1900	Serum albumin	9	183
		LOC101548172|XM_012935294.1 **|protein-9643**	***KLK1***	5	59
**P14**	22.9	ALB|XP_004681276.1 |protein-1900	Serum albumin	9	166
		KLK1|XP_004694095.1 **|protein-490**	***KLK1***	8	102
		LOC101548172|XM_012935294.1 **|protein-9643**	***KLK1***	16	97
**K14**	23.5	ALB|XP_004681276.1 |protein-1900	Serum albumin	9	176
		LOC101548172|XM_012935294.1 **|protein-9643**	***KLK1***	5	76
**A14**	24.6	ALB|XP_004681276.1 |protein-1900	Serum albumin	11	194
**A19**	31.8	ALB|XP_004681276.1 |protein-1900	Serum albumin	9	144
**L19**	33	ALB|XP_004681276.1 |protein-1900	Serum albumin	10	159

Table S4. Site-specific selection analysis of kallikreins.

	FUBAR[a]	MEME[b]	PAML[c] (M8)
**KLK outgroup clade**	**ω>1[d]: 1**<br>**ω<1[e]: 197**	**6**	**0**<br>**ω: 0.27**
**KLK4**	**ω>1[d]: 3**<br>**ω<1[e]: 86**	**1**	**0**<br>**ω: 0.28**
**KLK5**	**ω>1[d]: 1**<br>**ω<1[e]: 139**	**4**	**0**<br>**ω: 0.31**
**KLK6**	**ω>1[d]: 0**<br>**ω<1[e]: 99**	**2**	**0**<br>**ω*: 0.26**
**KLK7**	**ω>1[d]: 1**<br>**ω<1[e]: 113**	**4**	**0**<br>**ω: 0.35**
**KLK8**	**ω>1[d]: 5**<br>**ω<1[e]: 110**	**7**	**0**<br>**ω: 0.24**
**KLK9**	**ω>1[d]: 3**<br>**ω<1[e]: 145**	**6**	**0**<br>**ω: 0.15**
**KLK10**	**ω>1[d]: 5**<br>**ω<1[e]: 97**	**3**	**2**<br>**ω**: 0.27**
**KLK11**	**ω>1[d]: 0**<br>**ω<1[e]: 120**	**0**	**0**<br>**ω: 0.18**
**KLK12**	**ω>1[d]: 2**<br>**ω<1[e]: 92**	**5**	**0**<br>**ω**: 0.31**
**KLK13**	**ω>1[d]: 1**<br>**ω<1[e]: 137**	**2**	**0**<br>**ω: 0.19**
**KLK14**	**ω>1[d]: 1**<br>**ω<1[e]: 131**	**3**	**0**<br>**ω: 0.47**
**KLK15**	**ω>1[d]: 0**<br>**ω<1[e]: 127**	**11**	**0**<br>**ω: 0.30**
**KLK1 (1/2/3)**	**ω>1[d]: 2**<br>**ω<1[e]: 99**	**22**	**18**<br>**ω**: 0.55**

a: Fast Unconstrained Bayesian AppRoximation
b: Sites detected as experiencing episodic diversifying selection (0.05 significance) by the Mixed Effects Model Evolution (MEME)
c: Positively selected sites detected by the Bayes Empirical Bayes approach implemented in M8.
d: number of sites under pervasive diversifying selection at the posterior probability ≥0.9 (FUBAR)
e: Number of sites under pervasive purifying selection at the posterior probability ≥0.9 (FUBAR)
ω: mean dN/dS (*: p<0.05 and **: p<0.01)

Table S5. Branch model selection analysis of kallikreins.

Model	ω_a	Likelihood (l)	Number of Sites with $\omega > 1_b$
**Branch model (Two ratio)**	ω** = 0.46	-97395.029679	-
**Branch-site model A**	ω = 1.30	-95186.935886	39 (PP≥0.95)

a: dN/dS (*: p<0.05 and **: p<0.01)
b: Number of sites with ω > 1 under BEB approach with a posterior probability (PP) of more than or equal to 0.95.

Table S6. Branch-site unrestricted Statistical Test for Episodic Diversification (BUSTED) of kallikreins. BUSTED found evidence (LRT, p-value = ≤ .05) of gene-wide episodic diversifying selection in the selected test branches of the phylogeny. Therefore, there is evidence that at least one site on at least one test branch has experienced diversifying selection. FG – foreground; BG – background.

Model	log L	Branch	ω1	ω2	ω3
**Unconstrained model**	-96562.1	FG	0.01 (51.93%)	1.00 (45.32%)	2242.50 (2.74%)
		BG	0.01 (66.43%)	0.71 (29.75%)	591.83 (3.81%)
**Constrained model**	-96718.7	FG	0.00 (52.87%)	1.00 (0.22%)	1.00 (46.91%)
		BG	0.01 (66.77%)	0.72 (29.42%)	600.81 (3.81%)

Table S7. Adaptive Branch-Site Random Effects Likelihood analysis of kallikreins. Out of 461 branches, a total of 13 branches were found to have undergone episodic diversifying selection. All branches detected are found within the *KLK1* clade (which includes *Homo sapiens KLK1*, *KLK2* and *KLK3*). Four of the 13 branches detected are *KLK1* genes from *Solenodon paradoxus*, and three of these encode proteins detected in solenodon venom (highlighted red). A total of 49 branches were formally tested for diversifying selection. Statistical significance was computed using the Likelihood Ratio Test at a threshold of $p \leq 0.05$.

Branch	LRT	p-value	ω distribution over sites
Mus musculus **NM_010915.4** ***KLK1B4***	25.68	0.0000	ω_1 = 0.28 (93%) ω_2 = 33.8 (6.6%)
Solenodon paradoxus **9643** ***KLK1***	33.84	0.0000	ω_1 = 1.00 (93%) ω_2 = 398 (7.3%)
Solenodon paradoxus **3713** ***KLK1***	42.20	0.0000	ω_1 = 0.37 (87%) ω_2 = 79.6 (13%)
Canis familiaris **XM_014115208.2** ***KLK1***	59.78	0.0000	ω_1 = 0.00 (93%) ω_2 = 10000 (7.4%)
Homo sapiens **NM_001030047.1** ***KLK3***	103.84	0.0000	ω_1 = 0.06 (89%) ω_2 = 10000 (11%)
Homo sapiens **NM_001002231.2** ***KLK2***	71.94	0.0000	ω_1 = 0.16 (95%) ω_2 = 1630 (4.9%)
Solenodon paradoxus **7098** ***KLK1***	22.96	0.0001	ω_1 = 0.80 (95%) ω_2 = 10000 (5.1%)
Erinaceus europaeus **XM_016191784.1** ***KLK1***	24.27	0.0001	ω_1 = 0.72 (61%) ω_2 = 10000 (39%)
Felis catus **XM_019818671.2** ***KLK1***	21.64	0.0003	ω_1 = 0.83 (88%) ω_2 = 27.1 (12%)
Mus musculus **NM_010643.2** ***KLK1B24***	14.8611	0.0080	ω_1 = 0.00 (92%) ω_2 = 21.2 (8.5%)
Solenodon paradoxus **490** ***KLK1***	12.9580	0.0199	ω_1 = 1.00 (92%) ω_2 = 1090 (7.5%)
Homo sapiens **NM_002257.3** ***KLK1***	12.9790	0.0202	ω_1 = 0.22 (93%) ω_2 = 3330 (7.4%)
Mus musculus **NM_010114.2** ***KLK1B22***	12.6601	0.0225	ω_1 = 0.00 (86%) ω_2 = 11.4 (14%)

Table S8. Comparison of the activation and inactivation parameters for TE671 voltage gated sodium channels in the presence and absence of solenodon venom. *P* values were calculated using GraphPad Prism 7 by comparing conductance-voltage relationship curves using an extra sum-of-squares F test. N.S. not significant at P<0.05, n=5.

	**Parameter**	**Conditions**		***P value***
		Control	Solenodon venom (100 μg/mL)	
Activation	**$V_{50.act}$ (mV)**	-29.02 ± 0.87	-25.07 ± 0.54	0.0001
	***Slope* (mV)**	5.553 ± 0.75	4.68 ± 0.476	0.3286 (N.S)
Inactivation	**$V_{50.inact}$ (mV)**	-73.56 ± 0.87	-79.97 ± 1.14	0.0001
	***Slope* (mV)**	-7.86 ± 0.71	-8.87 ± 0.90	0.3788 (N.S)

SI Appendix Files

File S1. Detailed information on the peptide/protein spectrum matching for the shotgun, decomplexed, top-down and plasminogen-activating experiments.

File S2. A DNA sequence alignment of tetrapod kallikreins used to construct the Bayesian phylogeny displayed in SI Appendix Fig. S4.

File S3. An amino acid sequence alignment of tetrapod kallikreins used to construct the Bayesian phylogeny displayed in Fig. 4A.

File S4. Detailed information on the peptide/protein spectrum matching for the identification of plasminogen activating venom proteins.

SI References

1. Grigorev K, et al. (2018) Innovative assembly strategy contributes to understanding the evolution and conservation genetics of the endangered Solenodon paradoxus from the island of Hispaniola. *Gigascience* 7(6). doi:10.1093/gigascience/giy025.
2. Simão FA, Waterhouse RM, Ioannidis P, Kriventseva E V., Zdobnov EM (2015) BUSCO: assessing genome assembly and annotation completeness with single-copy orthologs. *Bioinformatics* 31(19):3210–3212.
3. Altschul SF, Gish W, Miller W, Myers EW, Lipman DJ (1990) Basic local alignment search tool. *J Mol Biol* 215(3):403–410.
4. Brandt AL, et al. (2017) Mitogenomic sequences support a north–south subspecies subdivision within *Solenodon paradoxus*. *Mitochondrial DNA Part A* 28(5):662–670.
5. Bernt M, et al. (2013) MITOS: Improved de novo metazoan mitochondrial genome annotation. *Mol Phylogenet Evol* 69(2):313–319.
6. Jurka J, et al. (2005) Repbase Update, a database of eukaryotic repetitive elements. *Cytogenet Genome Res* 110(1–4):462–7.
7. Holt C, Yandell M (2011) MAKER2: an annotation pipeline and genome-database management tool for second-generation genome projects. *BMC Bioinformatics* 12(1):491.
8. Cantarel BL, et al. (2008) MAKER: an easy-to-use annotation pipeline designed for emerging model organism genomes. *Genome Res* 18(1):188–96.
9. Campbell MS, Holt C, Moore B, Yandell M (2014) Genome Annotation and Curation Using MAKER and MAKER-P. *Current Protocols in Bioinformatics* (John Wiley & Sons, Inc., Hoboken, NJ, USA), pp 4.11.1-4.11.39.
10. Korf I (2004) Gene finding in novel genomes. *BMC Bioinformatics* 5(1):59.
11. Stanke M, Diekhans M, Baertsch R, Haussler D (2008) Using native and syntenically mapped cDNA alignments to improve de novo gene finding. *Bioinformatics* 24(5):637–644.
12. Eilbeck K, Moore B, Holt C, Yandell M (2009) Quantitative measures for the management and comparison of annotated genomes. *BMC Bioinformatics* 10(1):67.
13. Skinner ME, Uzilov A V, Stein LD, Mungall CJ, Holmes IH (2009) JBrowse: a next-generation genome browser. *Genome Res* 19(9):1630–8.
14. Buels R, et al. (2016) JBrowse: a dynamic web platform for genome visualization and analysis. *Genome Biol* 17(1):66.
15. Jones P, et al. (2014) InterProScan 5: genome-scale protein function classification. *Bioinformatics* 30(9):1236–1240.
16. Mitchell A, et al. (2015) The InterPro protein families database: the classification resource after 15 years. *Nucleic Acids Res* 43(D1):D213–D221.
17. Finn RD, et al. (2016) The Pfam protein families database: towards a more sustainable future. *Nucleic Acids Res* 44(D1):D279–D285.
18. Thomas PD, et al. (2003) PANTHER: a library of protein families and subfamilies indexed by function. *Genome Res* 13(9):2129–41.
19. Bateman A, et al. (2017) UniProt: the universal protein knowledgebase. *Nucleic Acids Res* 45(D1):D158–D169.
20. Conesa A, et al. (2005) Blast2GO: a universal tool for annotation, visualization and analysis in functional genomics research. *Bioinformatics* 21(18):3674–3676.
21. Nalbantsoy A, et al. (2017) Combined venom profiling and cytotoxicity screening of the Radde's mountain viper (Montivipera raddei) and Mount Bulgar Viper (Montivipera bulgardaghica) with potent cytotoxicity against human A549 lung carcinoma cells. *Toxicon* 135:71–83.
22. Barsnes H, Vaudel M (2018) SearchGUI: A Highly Adaptable Common Interface for Proteomics Search and de Novo Engines. *J Proteome Res* 17(7):2552–2555.
23. Zerbino DR, et al. (2018) Ensembl 2018. *Nucleic Acids Res* 46(D1):D754–D761.
24. Koumandou VL, Scorilas A (2013) Evolution of the Plasma and Tissue Kallikreins, and Their Alternative Splicing Isoforms. *PLoS One* 8(7):e68074.
25. Edgar RC (2004) MUSCLE: multiple sequence alignment with high accuracy and high throughput. *Nucleic Acids Res* 32(5):1792–7.
26. Ronquist F, et al. (2012) MrBayes 3.2: efficient Bayesian phylogenetic inference and model choice across a large model space. *Syst Biol* 61(3):539–42.
27. Guindon S, et al. (2010) New algorithms and methods to estimate maximum-likelihood phylogenies: assessing the performance of PhyML 3.0. *Syst Biol* 59(3):307–21.
28. Keane TM, Creevey CJ, Pentony MM, Naughton TJ, Mclnerney JO (2006) Assessment of methods for amino acid matrix selection and their use on empirical data shows that ad hoc assumptions for choice of matrix are not justified. *BMC Evol Biol* 6(1):29.
29. Yang Z (2007) PAML 4: phylogenetic analysis by maximum likelihood. *Mol Biol Evol* 24(8):1586–91.
30. Yang Z, Wong WSW, Nielsen R (2005) Bayes empirical bayes inference of amino acid sites under

positive selection. *Mol Biol Evol* 22(4):1107–18.
31. Murrell B, et al. (2012) Detecting individual sites subject to episodic diversifying selection. *PLoS Genet* 8(7):e1002764.
32. Murrell B, et al. (2013) FUBAR: a fast, unconstrained bayesian approximation for inferring selection. *Mol Biol Evol* 30(5):1196–205.
33. Yang Z (1998) Likelihood ratio tests for detecting positive selection and application to primate lysozyme evolution. *Mol Biol Evol* 15(5):568–73.
34. Yang Z, Nielsen R (1998) Synonymous and nonsynonymous rate variation in nuclear genes of mammals. *J Mol Evol* 46(4):409–18.
35. Smith MD, et al. (2015) Less is more: an adaptive branch-site random effects model for efficient detection of episodic diversifying selection. *Mol Biol Evol* 32(5):1342–53.
36. Kelley LA, Mezulis S, Yates CM, Wass MN, Sternberg MJE (2015) The Phyre2 web portal for protein modeling, prediction and analysis. *Nat Protoc* 10(6):845–58.
37. Ashkenazy H, Erez E, Martz E, Pupko T, Ben-Tal N (2010) ConSurf 2010: calculating evolutionary conservation in sequence and structure of proteins and nucleic acids. *Nucleic Acids Res* 38(Web Server issue):W529-33.
38. Ashkenazy H, et al. (2016) ConSurf 2016: an improved methodology to estimate and visualize evolutionary conservation in macromolecules. *Nucleic Acids Res* 44(W1):W344-50.
39. Kumar S, Stecher G, Tamura K (2016) MEGA7: Molecular Evolutionary Genetics Analysis Version 7.0 for Bigger Datasets. *Mol Biol Evol* 33(7):1870–1874.
40. Waterhouse AM, Procter JB, Martin DMA, Clamp M, Barton GJ (2009) Jalview Version 2--a multiple sequence alignment editor and analysis workbench. *Bioinformatics* 25(9):1189–91.
41. Aminetzach YT, Srouji JR, Kong CY, Hoekstra HE (2009) Convergent Evolution of Novel Protein Function in Shrew and Lizard Venom. *Curr Biol* 19(22):1925–1931.
42. Koludarov I, Aird SD (2019) Snake venom NAD glycohydrolases: Primary structures, genomic location, and gene structure. *PeerJ* In press. doi:10.7717/peerj.6154.
43. Paradis E, Schliep K (2018) ape 5.0: an environment for modern phylogenetics and evolutionary analyses in R. *Bioinformatics*. doi:10.1093/bioinformatics/bty633.
44. Revell LJ (2012) phytools: an R package for phylogenetic comparative biology (and other things). *Methods Ecol Evol* 3(2):217–223.
45. R Core Team (2018) R: a language and environment for statistical computing. Available at: https://www.r-project.org/.
46. Dubey S, Salamin N, Ohdachi SD, Barrière P, Vogel P (2007) Molecular phylogenetics of shrews (Mammalia: Soricidae) reveal timing of transcontinental colonizations. *Mol Phylogenet Evol* 44(1):126–137.
47. Springer MS, Murphy WJ, Roca AL (2018) Appropriate fossil calibrations and tree constraints uphold the Mesozoic divergence of solenodons from other extant mammals. *Mol Phylogenet Evol* 121:158–165.
48. Huelsenbeck JP, Nielsen R, Bollback JP (2003) Stochastic mapping of morphological characters. *Syst Biol* 52(2):131–58.
49. Debono J, et al. (2017) Coagulating colubrids: Evolutionary, pathophysiological and biodiscovery implications of venom variations between boomslang (Dispholidus typus) and twig snake (Thelotornis mossambicanus). *Toxins (Basel)* 9(5). doi:10.3390/toxins9050171.
50. Ainsworth S, et al. (2018) The paraspecific neutralisation of snake venom induced coagulopathy by antivenoms. *Commun Biol* In press.
51. Zietek BM, et al. (2018) Liquid chromatographic nanofractionation with parallel mass spectrometric detection for the screening of plasmin inhibitors and (metallo)proteinases in snake venoms. *Anal Bioanal Chem* 410(23):5751–5763.
52. Luther MA, et al. (1989) A muscle acetylcholine receptor is expressed in the human cerebellar medulloblastoma cell line TE671. *J Neurosci* 9(3):1082–96.
53. Gambale F, Montal M (1990) Voltage-gated sodium channels expressed in the human cerebellar medulloblastoma cell line TE671. *Brain Res Mol Brain Res* 7(2):123–9.
54. Ngum N (2019) Investigating the venom components of the Giant Indian centipede for Nav1.7 channel modifiers. Dissertation (University of Nottingham, UK).
55. Chaisakul J, et al. (2015) Prothrombin activator-like toxin appears to mediate cardiovascular collapse following envenoming by Pseudonaja textilis. *Toxicon* 102:48–54.
56. Mychajliw AM (2017) From the Pleistocene to the Anthropocene: fossils, genes, and the future of Caribbean mammals. Dissertation (Stanford University, USA).
57. Riaz T, et al. (2011) ecoPrimers: inference of new DNA barcode markers from whole genome sequence analysis. *Nucleic Acids Res* 39(21):e145.
58. Taylor PG (1996) Reproducibility of ancient DNA sequences from extinct Pleistocene fauna. *Mol Biol Evol* 13(1):283–285.
59. Kartzinel TR, et al. (2015) DNA metabarcoding illuminates dietary niche partitioning by African large

herbivores. *Proc Natl Acad Sci U S A* 112(26):8019–24.
60. Xiong M, et al. (2017) Molecular dietary analysis of two sympatric felids in the Mountains of Southwest China biodiversity hotspot and conservation implications. *Sci Rep* 7:41909.
61. Cooke SB, Dávalos LM, Mychajliw AM, Turvey ST, Upham NS (2017) Anthropogenic Extinction Dominates Holocene Declines of West Indian Mammals. *Annu Rev Ecol Evol Syst* 48(1):301–327.
62. Kita M, et al. (2004) Blarina toxin, a mammalian lethal venom from the short-tailed shrew Blarina brevicauda: Isolation and characterization. *Proc Natl Acad Sci U S A* 101(20):7542–7.
63. Kita M, et al. (2005) Purification and characterisation of blarinasin, a new tissue kallikrein-like protease from the short-tailed shrew Blarina brevicauda: comparative studies with blarina toxin. *Biol Chem* 386(2):177–82.
64. Duff MO, et al. (2015) Genome-wide identification of zero nucleotide recursive splicing in Drosophila. *Nature* 521(7552):376–9.
65. Fagerberg L, et al. (2014) Analysis of the human tissue-specific expression by genome-wide integration of transcriptomics and antibody-based proteomics. *Mol Cell Proteomics* 13(2):397–406.
